# Metal Cation Triggered Peptide Hydrogels and Their Application in Food Freshness Monitoring and Dye Adsorption

**DOI:** 10.3390/gels7030085

**Published:** 2021-07-07

**Authors:** Anna Fortunato, Miriam Mba

**Affiliations:** Dipartimento di Scienze Chimiche, Università degli Studi di Padova, via Marzolo 1, 35131 Padova, Italy; anna.fortunato.1@studenti.unipd.it

**Keywords:** peptide, hydrogel, metallogel, self-assembly, dye adsorption, sensing, organic amines, biogenic amines, food spoilage

## Abstract

Metal-ligand interactions have emerged as an important tool to trigger and modulate self-assembly, and to tune the properties of the final supramolecular materials. Herein, we report the metal-cation induced self-assembly of a pyrene–peptide conjugate to form hydrogels. The peptide has been rationally designed to favor the formation of β-sheet 1D assemblies and metal coordination through the Glu side chains. We studied in detail the self-assembly process in the presence of H^+^, Li^+^, Na^+^, K^+^, Ca^2+^, Ni^2+^, Cu^2+^, Zn^2+^, Cd^2+^, Co^2+^, Fe^3+^, and Cr^3+^ and found that the morphology and mechanical properties of the hydrogels are ion-dependent. Moreover, thanks to the presence of the metal, new applications could be explored. Cu^2+^ metallogels could be used for amine sensing and meat freshness monitoring, while Zn^2+^ metallogels showed good selectivity for cationic dye adsorption and separation.

## 1. Introduction

Supramolecular gels are soft materials originating from self-assembly in solution of a small molecule known as low molecular weight gelator (LMWG) [1,2,3]. Due to the reversible nature of the non-covalent forces that are involved, supramolecular gels are stimuli-responsive materials. They may actuate gel-to-sol transition, changes in morphology, color, viscosity, fluorescence, or conductivity in response to temperature, pH, ionic strength, ultrasounds, light, or mechanical forces [3,4,5,6,7,8]. A particular class of supramolecular gels are metallogels, in which gel formation is driven by metal-ligand interactions between metal ions and the organic LMWG [9,10,11,12]. Metals have been introduced not only to trigger self-assembly but also to tune the mechanical properties, catalytic activity [13,14], chemosensing [15], redox activity [16,17], luminescence [18,19], and self-healing properties [20,21,22].

Peptide based supramolecular hydrogels are of particular importance, as they find applications in tissue engineering [23,24], as biosensors [25,26,27], or as drug carriers [28,29,30]. On the other hand, peptide self-assembly has emerged also as an important tool for the design of bioinspired functional materials [31,32,33,34]. Most of the times, the formation of hydrogels is triggered by pH [35,36,37], salt concentration [38,39,40], or temperature changes. However, despite its potential, the investigation of peptide metallogels is still in its early stages [41]. Specific amino acids such as histidine, tyrosine, aspartic acid, or glutamic acid, possess side chains able to coordinate transition metal ions. Several studies demonstrated that metal cations can be used to trigger gel formation and tune morphology and mechanical properties in Phe–Phe dipeptide derivatives [39,42,43,44,45] and other short peptides [46,47,48]. Non-natural motives may be introduced into the peptide structure to favor metal binding [42,49,50]. Peptide-metallogels have shown new properties such as self-healing [42], catalytic activity [51], or redox activity [52].

The detection of volatile amines is of great interest as they are widely employed in the chemical, pharmaceutical, agricultural and food industries [53,54,55,56]. Biogenic amines (BA) (i.e., histamine, tyramine, putrescine, cadaverine, spermine, spermidine, tryptamine and serotonine) are produced and accumulated in food products as a result of fermentation or decomposition, and so they serve as freshness markers [57,58,59,60,61,62]. Foods can take many transit routes and experience different storage conditions before consumption, and thus “best before” or “use by” dates do not always ensure product freshness. For these reasons, in recent years, much effort has been exerted in order to develop smart packaging based on visual colorimetric sensors for food freshness monitoring in a consumer-friendly manner [63,64,65]. To facilitate the use in food packaging, a system able to detect BA vapors is desirable. In this context, supramolecular gels have been largely used for the fluorescent and colorimetric detection of organic volatile amines and biogenic amines [66,67,68,69], including some examples of amino acid based supramolecular gels reported by Xue and coworkers [70,71,72]. On the other hand, examples employing supramolecular metallogels are rare. Sengupta reported a Cu^2+^ metallogel that shows colorimetric changes in the presence of ammonia, diethylamine, trimethylamine, and pyridine vapors [73]. More recently, a Ni^2+^ metallogel was reported by Tang and coworkers [74] for the colorimetric detection of biogenic amines that could be used to monitor meat spoilage, while Gu and coworkers reported a Tb^3+^ gel able to detect organic amines at the sub-ppm level [75].

Organic dyes are an important class of organic compounds widely used in different industries, in particular in the textile industry. As such, they have become a critical source of water pollution and, since these pollutants are toxic and cause health disorders and perturbation of aquatic life, their removal from industrial waste water has come to be extremely important [76,77]. Different strategies have been developed for dye removal [77], among them, adsorption of dyes is an efficient low-cost and operationally simple approach [78]. Hydrogels and xerogels possess two intrinsic features that make them good candidates to be used as adsorbent materials for water remediation: a porous network and functional groups that can interact with guest molecules [79]. Consequently, peptide supramolecular gels have been extensively used as dye adsorption materials [80,81,82,83,84,85]. The use of peptide metallogels as dye adsorbents is almost unexplored, but in principle their superior mechanical properties should play a positive role. Qin and coworkers reported a Mg^2+^/L-glutamic acid dendron shrinkable gel capable of ionic dye adsorption and separation [86]. Ray and coworkers developed a Cu^2+^/peptide bolaamphiphile hydrogel that efficiently adsorbed various ionic and nonionic dyes [87].

**Figure 1:**
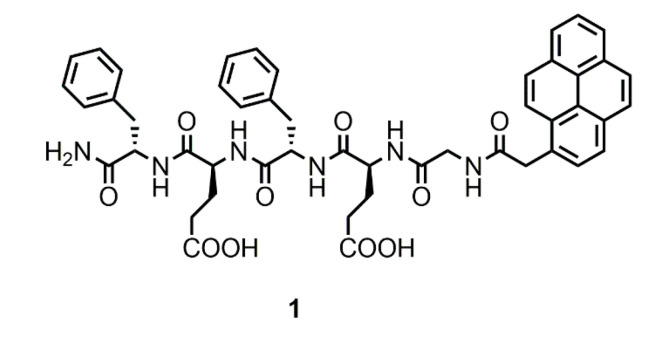
Structure of the pyrene–peptide conjugate **1**.

This work aims to further explore the potential of peptide metallogels. While most studies have focused on tuning morphology and mechanical properties, we are looking for new properties and applications derived from the presence of the metal. Herein, the metal-triggered formation of fluorescent hydrogels from pyrene–peptide conjugate **1** has been explored (Figure 1). The effect of different metal cations on gelation ability, morphology, mechanical and optical properties has been studied. We demonstrate that the Cu^2+^ triggered hydrogels are capable of amine sensing and can be used as food freshness indicators, while Zn^2+^ hydrogels were found to be able to selectively adsorb cationic dyes from their aqueous solutions.

## 2. Results and Discussion

### 2.1. Design and Synthesis of **1**

The peptide amphiphile **1** was designed to incorporate a fluorescent pyrene chromophore at the N-terminal position of a β-sheet forming pentapeptide containing two Glu residues in the sequence able to coordinate metal ions. Glu residues alternate with hydrophobic Phe residues, as this alternation is known to favor the formation of 1D β-sheet assemblies [88,89,90]. The synthesis was performed using standard Fmoc-based peptide synthesis protocols on a Rink amide resin. The identification of the product was assessed by NMR, FT-IR, and MS (Figures S1–S3, see supporting information for details).

### 2.2. pH-Triggered Gelation

**Figure 2:**
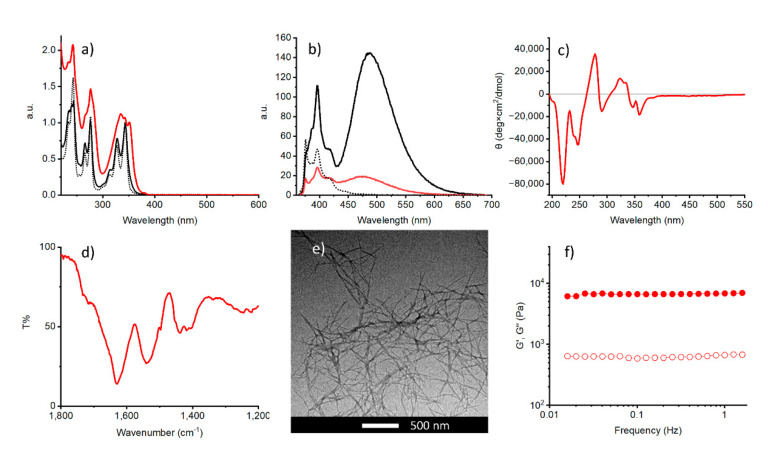
(**a**) UV-Vis absorption and (**b**) emission spectra of the pH-triggered gel (red line) and the aqueous solutions of **1** at 0.5% (solid black line) and 0.005% concentration (black dotted line); (**c**) ATR-FTIR, (**d**) CD spectrum, (**e**) TEM images and (**f**) frequency sweep experiment of the HCl triggered gel at 0.5% concentration, G′ is indicated with filled circles and G″ with open circles. ATR-IR and TEM were recorded using dried samples.

**1** dissolved in water at alkaline pH (pH ≈ 10) as a result of electrostatic repulsion between the deprotonated Glu side chains. At 0.005% concentration, the fluorescence spectrum of the solution was dominated by the characteristic emission of pyrene monomeric species [91]. However, at 0.5% concentration a new structureless band appears at 485 nm (Figure S4). The broader UV-Vis profile and nonsilent CD spectra of the 0.5% solution point to the formation of small aggregates at high concentrations (Figures S5 and S6). Acidification with a solution of HCl led to hydrogel formation within seconds. The minimum gelator concentration was 0.5%. The hydrogel was self-supporting and thermoreversible, with a Tgel of 107 °C. Thus, Tgel is higher than the boiling point of the solvent. This behaviour has been observed before in literature and can be ascribed to a remarkable stability of the 3D network [92,93,94,95]. The UV-Vis absorption spectrum of the gel showed the typical pyrene absorption band above 300 nm, but it was red-shifted, broader, and less structured when compared with the non-gelled sample (Figure 2a). These signatures suggest strong π–π interactions within the chromophores upon self-assembly. The emission spectrum of the gel was partially quenched and the vibrational peaks from to the monomeric species were still detected below 425 nm (Figure 2b). However, a new red-shifted broad centered at 475 nm appeared. This data further confirms the presence of aromatic π–π interactions. To get more insight, ATR-FTIR studies were performed on the xerogels. We examined the region between 1800 and 1430 cm^−1^ (Figure 2d). The peptide backbone gives rise to two bands in this region: the amide I band (~1650 cm^−1^), arising mainly from the C=O stretching, and the amide II band (~1550 cm^−1^), due to a combination of N–H in-plane bending and C–N stretching. It is well known that the secondary structure of peptides and proteins can be inferred from the position of the amide I [96]. Analysis of the ATR-IR spectrum showed a predominant Amide II band at 1630 cm^−1^, shifted to lower frequencies when compared to the non-aggregated sample (Figure S7). This signal is within the range expected for the β-sheet structure [97], and similar values have been reported for other short self-assembling peptides [37]. Moreover, in this region the C=O stretching of the Glu side chain is also observed. The protonated COOH gives rise to a single stretching at 1715 cm^−1^. The formation of extended β-sheet structures was confirmed by the presence of a strong negative signal at 215 nm in the circular dichroism spectrum (Figure 2c) [98,99,100]. Interestingly, Cotton effects were also evident between 300 and 400 nm, in the absorption region of the pyrene chromophore, suggesting a chiral arrangement of this chromophore within the supramolecular assembly [101].

The morphology of the gel was studied by transmission electron microscopy (TEM), which showed the formation of fibrous networks. Fibers were up to 800 nm long with a diameter of 21 ± 5 nm (Figure 2e).

The mechanical properties of the gel were studied by oscillatory rheology. As expected for viscoelastic materials, the storage modulus (G′) dominated over the viscous counterpart (G″) within the linear viscoelastic region (LVE) (Figure 2f), as expected for a gel material. In the tested LVE range, the storage and loss moduli were minimally sensitive to the frequency and did not cross each other. In strain sweep experiments the cross-over point (G′ = G″) was reached at 8% strain (Figure S8).

### 2.3. Metal Ion Triggered Gelation

A series of metal ions Li^+^, Na^+^, K^+^, Ca^2+^, Ni^2+^, Cu^2+^, Zn^2+^, Cd^2+^, Co^2+^, Fe^3+^, and Cr^3+^ were explored. Hydrogelation was studied by addition of different chloride salts to a 0.5% solution of **1** at basic pH. Different concentrations of salt were tested. Gelation triggered by monovalent cations required up to 12 equivalents of salt and took 5–10 min. On the other side, instant gel formation was observed upon addition of stoichiometric amounts of divalent and trivalent cations (Figure S9). Next, we varied the initial concentration of **1** and found that the minimum gelator concentration was reduced from 0.5% to 0.25% in the case of divalent and trivalent cations. We hypothesize that in the case of divalent and trivalent cations, metal complexation allows us to obtain a stable 3D network at lower concentrations. Similar trends have been reported before in the literature for other metal-triggered hydrogels [46].

To investigate the role of the different non-covalent forces involved in the formation of supramolecular hydro-metallogels, different spectroscopic techniques were employed. In the present case, UV-Vis and emission spectroscopies were used to assess the participation of π–π stacking interactions between pyrene chromophores. Meanwhile, ATR and CD spectroscopies were used to assess intermolecular hydrogen bond formation.

**Figure 3:**
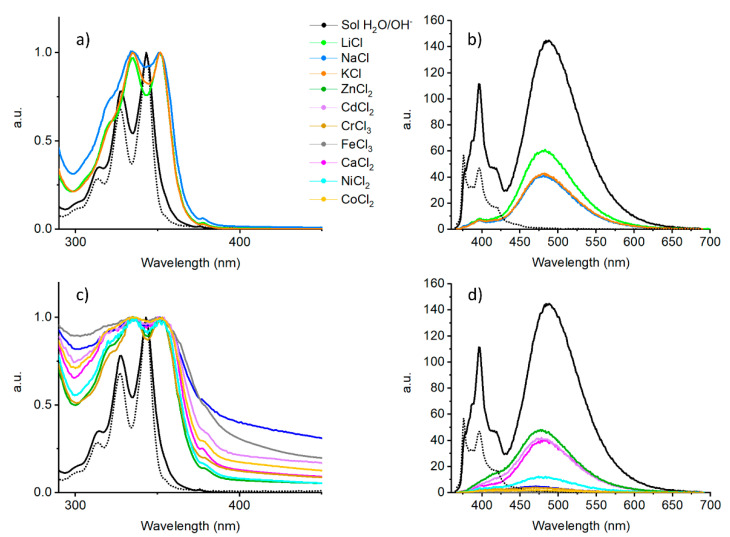
(**a**) Normalized UV-Vis absorption and (**b**) non-normalized emission spectra (λ_exc_ = 352 nm) of the monovalent cation triggered gels; (**c**) Normalized UV-Vis absorption and (**d**) non-normalized emission spectra (λ_exc_ = 352 nm) of the divalent and trivalent cation triggered gels. The data of the aqueous solutions of **1** at 0.5% (solid black line) and 0.005% concentration (black dotted line) have been included for comparison.

The profile of the UV-Vis absorption spectra of metal-triggered gels (Figure 3a,c) was in all cases broadened and red-shifted when compared to the non-aggregated state, as expected for the formation of supramolecular structures through strong π–π stacking interactions. This effect was more prominent in the case of salts bearing divalent and trivalent cations in which, in addition, a high scattering contribution was evident. The emission of metal-triggered hydrogels was strongly quenched (Figure 3b,d), in particular for Cr^3+^, Fe^3+^, Cu^2+^, Co^2+^, and Ni^2+^. The profile of the monomeric pyrene species was negligible in the emission spectra, which was indeed dominated by a broad red-shifted band.

In the ATR spectra of the xerogels (Figures S10 and S11), we observed the amide I band at 1632–1623 cm^−1^ shifted to lower wavenumber values when compared with the non-aggregated state. This shift indicates the formation of extended hydrogen bonding, in particular the position of the bands indicates the formation of β-sheet structures. The C=C stretching of the Phe side chain gives rise to the weak band at 1598 cm^−1^. In this region we also expected to see bands arising from the carboxylate anion: an asymmetric stretching (ν_as_(COO^−^)) at ~1560 cm^−1^ and a symmetric one (ν_as_(COO^−^)) at ~1404 cm^−1^. The broad band observed around 1540 cm^−1^ arises from the overlap of the amide II and the ν_as_(COO^−^). Next, three bands are found at circa 1453, 1440, and 1404 cm^−1^. The first two are caused by the CH bending of the side chains, the last one is the ν_as_(COO^−^). As expected, no stretching of the protonated COOH was detected. Thus in metallogels the Glu side chain is in the ionized form.

CD spectra were recorded for gels triggered by group I monovalent cations. The measurement was not possible when salts of divalent or trivalent cations were used to trigger gel formation, due to the high contribution of scattering. The CD spectra (Figure S12) were dominated by a negative peak at 214 nm, which suggests extensive β-sheet formation [98,99,100]. The profile of the Na^+^ triggered gel was much less intense in this region, probably due to the presence of bigger assemblies that affected the measurement. CD signals were also detected within the range of 300–400 nm, where only the pyrene contribution is expected. Thus, at least when monovalent cations are used, pyrene moieties take part in a chiral supramolecular assembly.

**Figure 4:**
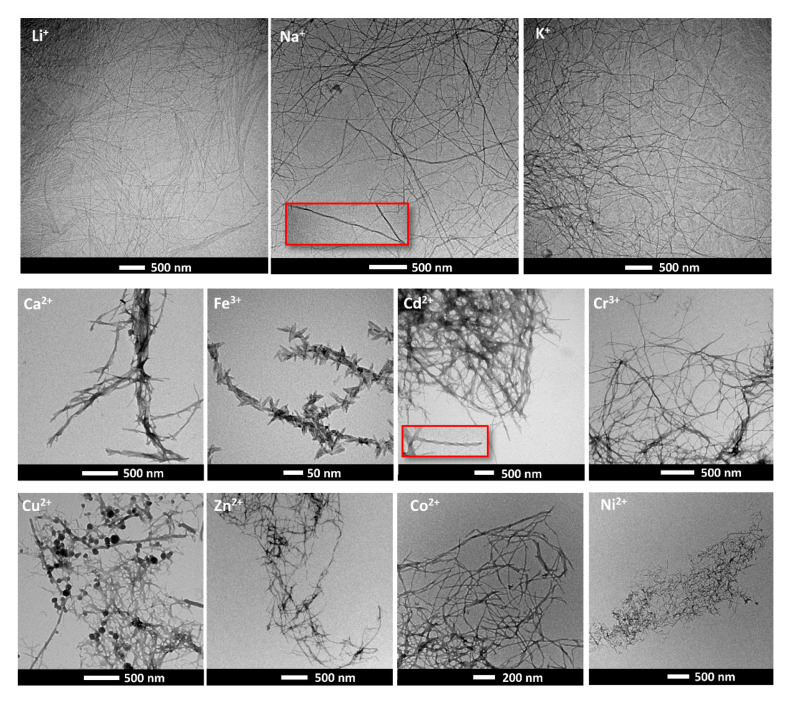
Transmission electron microscopy (TEM) images of the cation induced metallogels. Images were recorded in dry samples without the use of stain.

The morphology of the metallogels was studied by TEM (Figure 4 and Figures S13–S24). Monovalent cations gave rise to entangled fibrillar networks. Fibers were various microns long and, in the case of Na^+^ and K^+^, a helical twist of the fibers was observed. When divalent and trivalent cations are used, TEM images show in general the formation of shorter assemblies. Thus, it seems that when metal cations are used the growth of 1D structures is less effective. Three of the samples deserve special mention: the gels triggered by Cd^2+^, Cu^2+^, and Fe^3+^. As seen in Figure 4, superhelices were found in Cd^2+^ triggered gels. Fe^3+^ gave rise to the assemblies with the lowest aspect ratios. The supramolecular structures had a rod-like shape. On average, they measured 40 ± 8 nm in length with a diameter of 8 ± 2 nm. The inability of Fe^3+^ cation to form nanofibers has been ascribed before to strong peptide-metal interactions that inhibit nanofiber formation [43]. On the other hand, when Cu^2+^ was used, the TEM micrographs showed the coexistence of spherical structures, fibers, and worm-like micelles. We hypothesized that the difference in morphology may arise from different factors. First, while monovalent cations are expected to promote self-assembly by a simple screening of the charges, divalent and trivalent cations are on the other hand are able to form complexes. Metal complexation gives rise to more rigid structures and enhances cross-linking, but truncates the 1D growth through β-sheet hydrogen bonding, which explains the formation of shorter supramolecular structures. Second, the different coordination modes of the metal cations and the strength of the metal-ligand interaction will also affect the final morphology of the aggregates.

**Figure 5:**
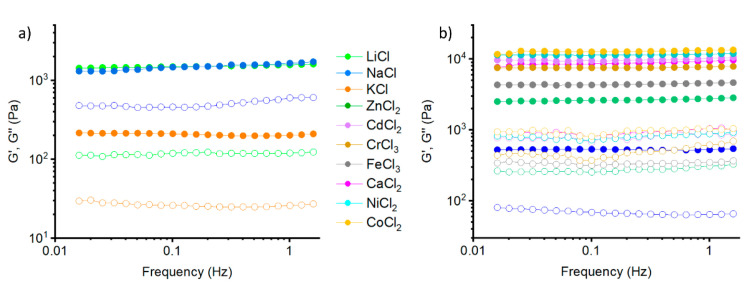
Frequency sweep experiments of metallogels triggered by (**a**) monovalent cations and (**b**) divalent and trivalent metal cations. G′ values are indicated with filled circles while G″ values are indicated with open circles.

The mechanical properties of the metallogels were examined by means of rheology. Frequency sweep measurements (Figure 5) showed that in all cases the storage modulus (G′) was higher than the loss modulus (G”) by at least one order of magnitude in the linear viscoelastic region (Table S1 of the SI). This behaviour confirms the gel nature of the materials. The G′ values in gels induced by monovalent cations were lower than the G′ of the pH-triggered gel, indicating less stiff gels. Strain sweep experiments, on the other hand, indicated that the gels break down at higher strain values (Figure S25).

Except for Cu^2+^, divalent and trivalent cations gave stiffer gels than monovalent cations and lower strain values were necessary to achieve the gel-to-sol transition (Table S1 of the SI). We hypothesized that metal complexation increases stiffness, however stiffer structures adapt less to strain changes and so gel failure is achieved at lower strain values. When compared to the HCl-gel, divalent and trivalent cations required lower strain deformations for gel failure (except for Cu^2+^) (Figure S26). On the other side, only Co^2+^ and Ni^2+^, which according to the Irwin-Williams series are predicted to possess higher complex stability, had significant a higher G′ than the pH-triggered gel. The Cu^2+^ triggered gel is a case apart. It showed the lowest G′ value of the series. This behaviour is a consequence of the particular morphology of the hydrogel. A weaker 3D network is expected, as spherical assemblies do not favour an effective cross-linking.

### 2.4. Detection of Amines and Meat Freshness Monitoring

**Figure 6:**
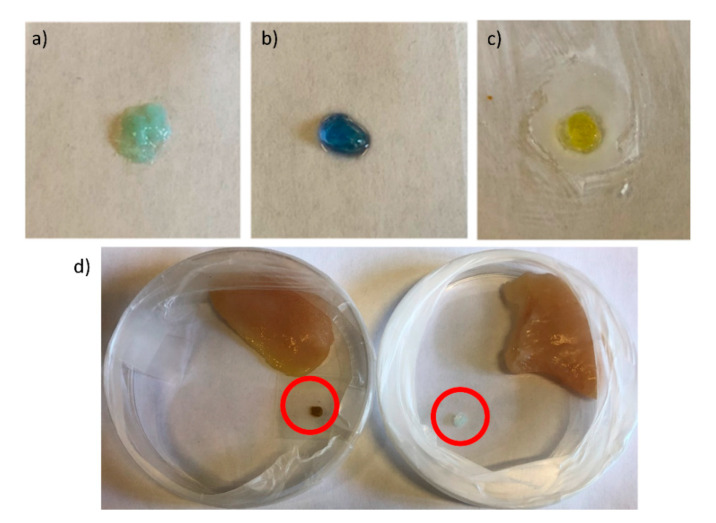
Images of the **1**-Cu^2+^ gel (**a**) as prepared, (**b**) after exposure to ammonia vapors, and (**c**) after exposure to HCl vapors, (**d**) colorimetric responses of the **1**-Cu^2+^ gel when exposed to chicken meat stored at 25 °C (left) and 4 °C (right).

We investigated the ability of the **1**-Cu^2+^ metallogel to detect volatile amine vapors. We expected to observe colorimetric changes upon exposure to amines due to a change in the coordination environment of the copper ions, which are known to coordinate amines to give blue complexes. For sensing experiments, films were obtained on a glass slide by drop-casting a solution of **1** and careful addition of CuCl_2_ solution. The obtained hydrogel was light blue. The hydrogel was then exposed to amine vapors in a closed chamber. In particular, the response to ammonia, methylamine, diethylamine and triethylamine was investigated. The hydrogel changes from light blue to dark blue color in the presence of ammonia and methylamine, while it changes to violet color when exposed to diethylamine and trimethylamine (Figure S27). The gel recovered its initial color after exposure to air for several hours or upon brief exposure to HCl vapors. In the presence of air, the amine-coordinated gel reverted to the original state, inducing a rearrangement of the copper ion coordination. Encouraged by these results, we decided to investigate the potential of the **1**-Cu^2+^ gel in monitoring chicken breast spoilage. To do so, a controlled spoilage environment was used as a model of packaging. A piece of chicken breast and a piece of metallogel were introduced to a Petri dish while avoiding contact with each other, and then the dish was sealed. Two experiments, at 25 °C and 4 °C, were performed (Figure 6). When the sample was kept at 4 °C, no color changes were detected even after three days. On the other hand, when the sample was stored at 25 °C the metallogel changed its color from light blue to dark brown in only 12 h, indicating meat degradation. This is, as far as we know, the first example of a peptide-metallogel sensor for monitoring food spoilage.

### 2.5. Selective Cationic Dye Adsorption

**Figure 7:**
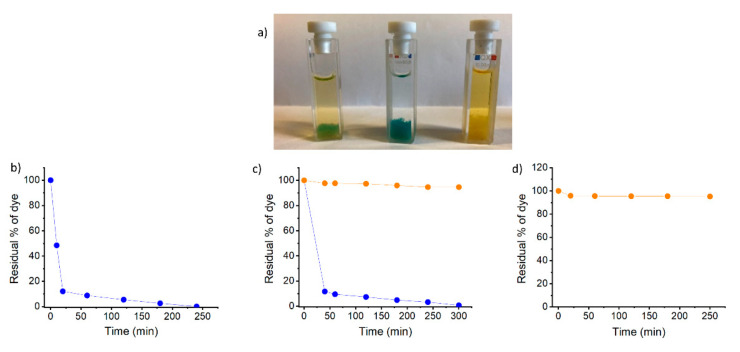
(**a**) Image of cuvettes containing **1**-Zn^2+^ xerogels upon dye adsorption, from left to right methylene blue mixed with methyl orange, methylene blue, and methyl orange. Dye absorption abilities of the **1**-Zn^2+^ xerogel: (**b**) Evolution over time of the residual concentration (%) of methylene blue (668 nm), (**c**) Evolution over time of the residual concentration (%) of dye in a 1:1 mixture of methylene blue (668 nm, blue dots) and methyl orange (463 nm, orange dots) (**d**) Evolution over time of the residual concentration (%) of methyl orange (463 nm).

As pointed out in the Introduction, dye adsorption is a simple and efficient way to remove dyes from wastewater. We investigated the dye removal ability of our hydrogels using two common dyes: methylene blue and methyl orange. First, a qualitative test was performed in both pH and cation triggered hydrogels. To do so, 0.75 mL of a 15 mg/L solution of methylene blue was carefully added to a vial containing 0.25 mL of the corresponding hydrogels obtained at 0.5% concentration. The samples were allowed to stand undisturbed at room temperature. We observed that gels triggered by H^+^, Ca^2+^, Fe^3+^, and monovalent cations fall apart upon dye absorption. On the other hand, gels triggered by divalent transition metal cations remain intact (Figure S28). Among them, the Zn^2+^ triggered hydrogel showed the fastest dye adsorption (Figures S29–S31) and so we continued the dye removal studies with it. To facilitate adsorbent separation and recovery, and to avoid diffusion effects, we lyophilized the **1**-Zn^2+^ metallogel and performed our studies on the xerogel. Adsorption studies were performed using the cationic dye methylene blue, the anionic dye methyl orange, and a mixture of both (Figure 7 and Figures S32–S34). In a typical experiment, 4 mg of xerogel were introduced to a vial together with 2.7 mL of a 10 mg/mL aqueous solution of the dye. After 30 min, it was evident that the xerogel was able to completely adsorb methylene blue, as the water appeared colorless while the xerogel was dark blue. On the other side, methyl orange was not adsorbed by the xerogel and when a mixture of both dyes was used, the xerogel became blue while the aqueous phase remained orange, indicating the selective adsorption of the cationic dye. The process was also monitored by UV-Vis spectroscopy. The 668 nm and 463 nm wavelengths were used to monitor methylene blue and methyl orange, respectively. As show in Figure 7, the removal efficiency for methylene blue was remarkable. The concentration of the dye in the solution decreased quickly and 89% of the dye was removed after only 20 min, while methyl orange was not removed from water. In a mixture, the intensity of the absorption of methylene blue decreases with time, while the absorption of methyl orange remains unaffected. The results indicate that the **1**-Zn^2+^ xerogel selectively removes cationic dyes from water. Selectivity is related to the attractive electrostatic interaction between the deprotonated Glu side chains of the peptide and the positive charge of the thiazine dye.

## 3. Conclusions

In summary, we have described the cation-induced self-assembly of a pyrene–peptide conjugate. The self-assembly process was studied at acidic pH and at alkaline pH by the addition of monovalent, divalent, and trivalent cations. In particular, the interaction of the anionic Glu side chain with the metal cation was exploited to induce self-assembly. Our studies also demonstrated that β-sheet hydrogen bonding and π–π interactions between pyrene moieties contribute to the formation of the supramolecular assemblies. We found that the morphology and mechanical properties of the resulting hydrogels are cation-dependent. Long, flexible fibrillar structures were obtained when gelation was triggered by pH or addition of monovalent cations. Shorter, more rigid structures were obtained when divalent and trivalent cations were used. The Cu^2+^ metallogel had a particular morphology, and the coexistence of fibers, warm-like micelles and spherical structures was found. This metallogel was used for the colorimetric detection of organic volatile amines, and we demonstrated its utility in monitoring meat spoilage. Moreover, thanks to the particular porosity and mechanical properties, the Zn^2+^ metallogel showed a good ability for selective dye adsorption.

## 4. Materials and Methods

### 4.1. General Methods

ESI-MS experiments were performed on an ESI-TOF MarinerTM BiospectrometryTM Workstation of Applied Biosystems by flow injection analysis using methanol/1% formic acid as a mobile phase. ^1^H, ^13^C, and 2D NMR were recorded at 289 K on a Bruker Avance III 300 spectrometer using the partially deuterated solvent as internal reference. Chemical shifts (δ) are expressed in parts per million (ppm). The multiplicity of a signal is indicated as: s (singlet), d (doublet), t (triplet), dd (doublet of doublets), dt (doublet of triplets), td (triplet of doublets), q (quartet), and m (multiplet). The acronym “br” indicates a broadened signal. FT-IR absorption spectra were recorded on an FT-IR Perkin-Elmer, model 1720X spectrophotometer, in KBr disk, at a nominal resolution of 2 cm^−1^, averaging 100 scans. In the case of hydrogels, IR spectra were recorded using the xerogels. UV-Vis absorption spectra were recorded on a Varian Cary 50 spectrophotometer at 25 °C. All spectra are baseline corrected. A rectangular cell with detachable windows and optical path of 0.02 cm was used for the analysis of gelled samples. For non-gelled samples, a reduce volume quartz cell with 1 cm or 0.1 cm optical path was used. General methodology for gel samples: gels were prepared in a glass vial; a small amount was transferred to the sample chamber and the cell was closed with the top window taking care not to form bubbles. Emission spectra were recorded on a Varian CaryEclipse spectrophotometer at 25 °C. A quartz cell with an optical path of 10 × 4 mm and volume 1400 μL was used for gel samples. A quartz cell with optical path of 10 × 10 mm and volume 3 mL was used for the solutions. General methodology for gel samples: Gels were prepared in a glass vial and transferred to the cuvette without amendment such as dilution. CD spectra were recorded on a Jasco J-1500 instrument at 25 °C and were baseline corrected. Every CD spectrum was obtained as an average of 32 or 64 measurements. The spectra are expressed in terms of total molar ellipticity (deg·cm^2^·dmol^−1^). A rectangular cell with detachable windows and optical path of 0.02 cm was used for the analysis of gelled samples. For non-gelled samples, a reduce volume quartz cell with 1 cm or 0.1 cm optical path was used. General methodology for gel samples: gels were prepared in a glass vial; a small amount was transferred to the sample chamber and the cell was closed with the top window taking care not to form bubbles. Transmission electron microscopy (TEM) images were recorded on a Jeol 300PX instrument. A glow discharged carbon coated grid was floated on a small drop of solution and the excess was removed by using #50 hardened Whatman filter paper. Samples of the gels were prepared in two different ways: (a) by dropping a small amount of gel into a glow discharged carbon coated grid and removing the excess gel with #50 hardened Whatman filter paper; (b) gels were diluted prior to analysis, a small amount of each sample has been deposited directly on a glow discharged carbon coated grid and no staining has been used. The excess has been removed by #50 hardened Whatman filter paper. The images were analysed with the ImageJ program. Rheological analyses were carried out on a Kinexus Lab+ rheometer with a parallel plate geometry. Hydrogel samples were prepared on a total volume of 2 mL and immediately transferred onto the plate. An anti-evaporation chamber was used to prevent drying of the samples and temperature was set at 25 °C. Frequency sweep tests were carried out between 0.6–0.0016 Hz at a constant strain. Strain measurements were carried out between 0.01–110% at a constant frequency of 1 Hz.

### 4.2. Synthesis of **1**

Compound **1** was synthesized using standard solid phase 9-fluorenylmethoxycarbonyl (Fmoc) chemistry on Rink Amide resin. When not in use, the resin was dried and stored in a freezer with the amino-terminus Fmoc-protected. The MBHA Rink Amide resin was purchased from Irish Biotech (Marktredwitz, Germany) (commercial loading 0.68 mmol/g); *O*-Benzotriazole-*N*,*N*,*N*′,*N*′-tetramethyluronium hexafluorophosphate (HBTU), 1-Hydroxybennzotriazole hydrate (HOBt), *N*,*N*-Diisopropylethylamine (DIPEA), Piperidine, trifluoroacetic acid (TFA), triisopropylsilane (TIPS), Fmoc- amino acids and solvents were purchased from Sigma-Aldrich-Merck (Darmstadt, Germany). SPPS was performed in a standard vessel for manual SPPS equipped with a glass frit and two outlets. The stirring was achieved by bubbling nitrogen from below, thus all the steps have been carried out under N_2_ flux. A washing step implies 1 min of stirring and then removal of solvent. In general, 10 mL of solvent must be used for 1 g of resin. The resin was prepared by dumping 1g of resin into the SPPS vessel and then 10mL of dimethylformamide (DMF) were added for resin swelling and stirred gently for 30 min. Fmoc deprotection was performed by mixing the resin in a piperidine/DMF (2:8) solution for 15 min (×2, then washing with DMF (×). For amino acid couplings we used the following protocol: 4.0 eq. (relative to the resin loading) of Fmoc-protected amino acid were activated externally with 3.9 eq. of HBTU, 3.9 eq. of HOBt and 12 eq. of DIPEA in DMF. This mixture was then added to a peptide chamber containing the Rink Amide resin and mixed for 3 h. All coupling and deprotection steps were monitored using a Kaiser test on a few resin beads that were removed from the peptide chamber after drying with dichloromethane (DCM). If necessary, the coupling step was repeated. The coupling with the pyrene functionalized core was performed using 2 eq. of 1-Pyreneacetic acid, 1.9 eq. of HBTU and HOBt and 6 eq. of DIPEA. The reaction was performed for 2 h. The solvent was removed and the resin was washed with DMF (3 × 10 mL), DCM (3 × 10 mL), and DMF (2 × 10 mL). Cleavage from the resin and removal of side-chain protecting groups was accomplished by stirring the resin with 10 mL of TFA, water, and TIPS (95:2.5:2.5) for 3 h. The solvents were collected in a flask and the resin (that eventually turned red) was washed with DCM (3 × 10 mL). Solvents collected were concentrated in rotavapor (a potassium hydroxide trap was used) to the half. DCM was added and the volatiles were evaporated again. The process was repeated three times, after which the solvents were evaporated to dryness. The product was precipitated from cold diethyl ether and the precipitated peptide was isolated by centrifugation and lyophilized. A white powder was obtained (89% yield).

ESI-MS: [M+H]^+^, calculated for C_48_H_49_N_6_O_10_ 869.34, found: 869.3. [M+Na]^+^ calculated for C_48_H_48_N_6_NaO_10_ 891.34, found 891.4.

^1^H-NMR (DMSO-d6, 300 MHz): δ 12.02 (bs, COOH), 8.44–8.30 (m, 2H), 8.28–7.89 (m, 11H), 7.78 (d, *J* = 8.0 Hz, 1H), 7.30 (s, 1H, NH_2_), 7.11 (dd, *J* = 21.5, 4.5 Hz, 10H, Ar of Phe), 7.04 (s, 1H, NH_2_), 4.44–4.36 (m, 2H, H_α_, Phe), 4.25–4.16 (m, 4H, overlapping signal H_α_ Glu and Gly), 3.72–3.71 (d, *J* = 4.1 Hz, 2H, Pyrene), 3.01–2.88 (m, 2H, H_β_, Phe), 2.82–2.63 (m, 2H, H_β_, Phe), 2.20–2.09 (m, 4H, m, H_γ_, Glu), 1.86–1.58 (m, 4H, H_β_, Glu) ppm.

^13^C-NMR (DMSO-d6, 75 MHz) δ 174.79, 174.73, 173.42, 171.91, 171.86, 171.47, 169.85, 138.54, 138.50, 131.63, 131.52, 131.18, 130.58, 129.97, 129.85, 129.49, 128.84, 128.78, 128.19, 128.08, 127.64, 126.98, 125.87, 125.72, 125.56, 124.90, 124.73, 54.73, 54.49, 53.06, 52.72, 43.10, 38.30, 37.72, 30.83, 28.13, 20.02.

FT-IR (KBr):$\tilde{\nu }$ (cm^−1^) = 3403, 3311, 3030, 2927, 1737, 1722, 1659, 1652, 1615, 1540, 14,898, 1453, 1443, 1409, 1272, 1185, 1170, 846, 792, 744, 699, 646, 496.

### 4.3. Gel Preparation

pH triggered gelation: a known amount of **1** was introduced to a 4 mL vial. Then, 900 μL of milliQ water was added. NaOH 1M was added in portions of 10 μL until a clear solution through sonication was obtained followed by addition of water to a final volume of 1 mL. Then, HCl 1M was added in portions. Gel formation was assessed by the vial inversion test.

Alkali metal salt induced gelation: a known amount of **1** was introduced to a 4 mL vial. Then, 900 μL of milliQ water was added. NaOH 1M was added in portions of 10 μL until a clear solution was obtained (sonication) followed by addition of water to a final volume of 1 mL. Then, a solution of alkali salt 1M was added in portions of 10 μL until gel formation was observed. Gel formation was assessed by the vial inversion test.

Bi-and-trivalent metal salts induced gelation: a known amount of **1** was introduced to a 4 mL vial. Then, 900 μL of milliQ water was added. NaOH 1M was added in portions of 10 μL until a clear solution was obtained (sonication) followed by addition of water to a final volume of 1 mL. Then, a solution of salt 0.05 M was added at a 1:1 ratio with compound 1. Gel formation was assessed by the vial inversion test.

The temperatures of gel-to-sol transition were determined as follows: 1 mL of gel at the appropriate concentration was prepared in a clear glass vial. The vial was sealed, placed tilted on thermostated oil bath, and heated at a rate of 0.5 °C/min. The temperature at which the gel starts to break down was defined as Tgel. The measurement was repeated at least twice, and the average value is reported. Tgel values were found to be almost unaltered within a difference of 1–2 °C after two heating–cooling cycles.

## Data Availability

The data presented in this study are available in the article.

## Supplementary Materials

The following are available online at https://www.mdpi.com/article/10.3390/gels7030085/s1, Figure S1: ESI-MS of compound **1**, Figure S2: (a) ^1^H NMR (DMSO-d6, 300 MHz) of **1**, (b) ^13^C-NMR (DMSO-d_6_, 300 MHz) of **1**, Figure S3: FT-IR spectrum of **1** (KBr disk), Figure S4: Non-normalized emission spectra of **1** in basic water at 0.5% (solid line) and at 0.005% (dotted line), Figure S5: Normalized absorption spectra of **1** in basic water at 0.5% (solid line) and at 0.005% (dotted line), Figure S6: CD spectra of **1** in basic water at 0.5% (solid line) and at 0.005% (dotted line), Figure S7: ATR-FTIR spectrum of **1** (solid line) and the lyophilized deprotonated sample (dotted line), Figure S8: Amplitude sweep experiment of the **1**-HCl gel, Figure S9: Picture of vials containing the hydrogels obtained with the different triggers, Figure S10: ATR-FTIR spectra of the monovalent cation induced hydrogels recorded on the lyophilized samples, Figure S11: ATR-FTIR spectra of divalent and trivalent cation induced hydrogels recorded on the lyophilized samples, Figure S12: CD-spectra of the monovalent cation induced hydrogels, Figures S13–S24: TEM images of the hydrogels, Figures S25 and S26: Amplitude sweep experiments of metal-induced hydrogels; Table S1: Average value of G′ and G″ and the flow point, Figure S27: Colorimetric responses of the **1**-Cu^2+^ gel to methylamine, diethylammine, and triethylammine, Figure S28: Photo of cuvettes containing hydrogels and adsorbed methylene blue, Figure S29: Absorption spectra of a 15 mg/L solution of methylene blue in the presence of **1**-HCl hydrogel, Figure S30: Absorption spectra of a 15 mg/L solution of methylene blue in the presence of **1**-Na+ hydrogel, Figure S31: Absorption spectra of a 15 mg/L solution of methylene blue in the presence of **1**-Zn^2+^ hydrogel, Figure S32: Absorption spectra of a 10 mg/L solution of methylene blue in the presence of **1**-Zn^2+^ xerogel, Figure S33: Absorption spectra of a 10 mg/L solution of methyl orange in the presence of **1**-Zn^2+^ xerogel, Figure S34: Absorption spectra of a mixture of methylene blue and methyl orange, 5 mg/mL, in the presence of **1**-Zn^2+^ xerogel.
