# Supplementary material for: Metal Cation Triggered Peptide Hydrogels and Their Application in Food Freshness Monitoring and Dye Adsorption
Source: Gels. 2021 Jul 7;7(3):85. doi: 10.3390/gels7030085 (PMC8293139; doi:10.3390/gels7030085)
Supplement: Supplementary file 1 [file gels-07-00085-s001.zip › gels-1268216-supplementary.pdf]

Supporting information

Metal-cation triggered peptide hydrogels and their application in the detection of biogenic amines and dyeadsorption

Anna Fortunato [1], Miriam Mba [1,*]

General methods **3**

Synthetic Procedure and Characterization of 1 .... **5**

Synthesis of **1** **5**

S1: ESI-MS spectra of **1** 7

S2 a and b: ^{1}H-NMR and ^{13}C-NMRspectra of **1** 8

S3: FT-IR spectra of **1** 9

S4: UV-Vis absorption spectra of **1** 10

S5: UV-Vis emission spectra of **1** 10

S6: CD spectrum of **1** 11

S7: ATR-FTIR spectra of **1** 11

Preparation and Characterization of Gels **12**

Preparation of gels 12

S8: Amplitude sweep of **1-HCl** gel 12

S9-S10: ATR-FTIR of xerogels 13-14

S11: CD spectra of monovalent hydrogels 14

S12-S23: TEM images 15-20

S24-S25: Amplitude sweep 21

T1: Table of G', G'' and flow point 21

S26: Colorimetric responses of the 1-Cu^{2+} hydrogel to methylamine, diethylamine and trimethylamine........21

S27: UV-Vis absorption of methylene blue with **1-Zn^{2+}** xerogel 23

S28: UV-Vis absorption of methyl orange with **1-Zn^{2+}** xerogel 23

S29: UV-Vis absorption of methylene blue mixed with methyl orange with **1-Zn^{2+}** xerogel 24

S30: Photo of **1-HCl**, **1-Na^{+}** and **1-Zn^{2+}** hydrogels in presence of methylene blue........24

S31: UV-Vis absorption of methylene blue with **1-HCl** hydrogel 24

S32: UV-Vis absorption of methylene blue with **1-Na^{+}** hydrogel 25

S33: UV-Vis absorption of methylene blue with **1-Zn^{2+}** hydrogel 25

GENERAL METHODS

- Mass Spectrometry

ESI-MS experiments were performed with a ESI-TOF MarinerTM BiospectrometryTM Workstation of Applied Biosystems by flow injection analysis using methanol with formic acid (1 %) as mobile phase.

- NMR Spectroscopy

^{1}H, ^{13}C and 2D NMR were recorded at 289 K on a Bruker Avance III 500 spectrometer using the partially deuterated solvent as internal reference. Deuterated DMSO has been used as solvent (99.9% d_6, Sigma Aldrich). Chemical shifts (δ) are expressed in parts per million (ppm). The multiplicity of a signal is indicated as: s (Singlet), d (Doublet), t (Triplet), dd (Doublet of doublets), dt (Doublet of triplets), td (Triplet of doublets), q (Quartet) and m (Multiplet). The acronym "br" indicates a broadened signal. The spectral width for ^{1}H-NMR is from 0ppm to 14ppm, whereas the ^{13}C-NMR spectral width is from 0 ppm to 210 ppm.

- FT-IR

FT-IR absorption spectrum were recorded with a FT-IR Perkin-Elmer, model 1720X spectrophotometer, in KBr disk, at a nominal resolution of 2 cm^{-1}, averaging 100 scans.

- UV-Vis Spectroscopy

UV-Vis absorption spectra were recorded with a Varian Cary 50 spectrophotometer at 25°C. All spectra are baseline corrected. A rectangular cell with detachable windows (Hellma) and optical path of 0.02 cm (Hellma) was used for the analysis of gelled samples. For non-gelled samples a reduce volume quartz cell with 1 cm or 0.1 cm optical path was used.
General methodology for gel samples: gels were prepared in a glass vial; a small amount was transferred to the sample chamber and the cell was closed with the top window taking care of not forming bubbles.

- Emission Spectroscopy

Emission spectra were recorded in a Varian CaryEclipse spectrophotometer at 25°C. A quartz cell with optical path of 10x4 mm and volume 1400 μL was used for gel samples. A quartz cell with optical path of 10x10 mm and volume 3 mL was used for solutions.
General methodology for gel samples: Gels were prepared in a glass vial and transferred to the cuvette without amendment such as dilution.

- Circular Dichroism Spectroscopy

CD spectra were recorded on a Jasco J-1500 instrument at 25°C and were baseline corrected. The spectra are expressed in terms of total molar ellipticity (deg·cm^2·dmol^{-1}). For non-gelled samples a reduce volume quartz cell with 1 cm or 0.1 cm optical path was used.

General methodology for gel samples: gels were prepared in a glass vial; a small amount was transferred to the sample chamber and the cell was closed with the top window taking care of not forming bubbles.

- TEM

Transmission electron microscopy (TEM) images were recorded with a Jeol 300PX instrument. A glow discharged carbon coated grid was floated on a small drop of solution and excess was removed by using #50 hardened Whatman filter paper. Samples of the gels were prepared in two different ways: a) by dropping a small amount of gel into a glow discharged carbon coated grid and removing excess of gel with #50 hardened Whatman filter paper; b) gels were diluted prior to analysis, a small amount of each sample has been deposited directly on a glow discharged carbon coated grid and no staining has been used. The excess has been removed by #50 hardened Whatman filter paper. The images obtained have be analysed with ImageJ program.

SYNTHETIC PROCEDURES

Compound **1** was synthesized using standard solid phase 9-Fluorenylmethoxycarbonyl (Fmoc) chemistry on Rink amide resin. When not in use the resin was dried and stores in freezer with the amino-terminus Fmoc-protected. The MBHA Rink Amide Resin was purchased from Irish Biotech (commercial loading 0.68 mmol/g); O-Benzotriazole-N,N,N',N'-tetramethyluronium hexafluorophosphate (HBTU), 1-Hydroxybennzotriazole hydrate (HOBt), N,N-Diisopropylethylamine, Piperidine, all the amino acids and solvents were purchased from Sigma-Aldrich. SPPS was performed in a standard vessel for manual SPPS equipped with a glass frit and two outlets. The stirring was achieved by bubbling nitrogen from below, thus all the steps have been carried out under N_2 flux. A washing step implies 1 min of stirring and then removal of solvent. In general, 10 mL of solvent must be used for 1 gram of resin. The resin was prepared dumping 1g of resin into the SPPS vessel and then 10mL of DMF were added for resin swelling and stirred gently for 30 minutes. For each amino acid coupling step (i), Fmoc deprotection was performed by mixing the resin in a piperidine/DMF (2:8) solution for 15 minutes (x2), then washing with DMF (x3). For all of the amino acid couplings we used the following protocol: 4.0 eq. (relative to the resin loading) of Fmoc-protected amino acid were activated externally with 3.9 eq. of HBTU, 3.9 eq. of HOBt and 12 eq. of DIPEA in DMF. This mixture was then added to a peptide chamber containing the Rink amide resin and mixed for 3 hours. All coupling and deprotection steps were monitored by performing a Kaiser test on a few resin beads which were removed from the peptide chamber after drying with DCM. If necessary, the coupling step was repeated. The deprotection activation and coupling steps were repeated until the desiderated structure was obtained.

The coupling with pyrene functionalized core (ii) was performed using 2 equiv of 1-Pyreneacetic acid, 1.9 equiv of HBTU and HOBt and 6 equiv of DIPEA. The reaction was performed for 2 hours. The solvent was removed and the resin was washed with DMF (3 x 10 mL), DCM (3 x 10 mL) and DMF (2 x 10 mL).

Cleavage from the resin and removal of side-chain protecting groups (iii) was accomplished by stirring the resin with 10 mL of TFA, water and TIPS (95:2.5:2.5) for 3 hours. The solvents were collected in a flask and the resin (that eventually turned red) was washed with DCM (3 x 10 mL). Solvents collected were concentrated in rotavapor (a potassium hydroxide trap was used) to the half. DCM was added and the volatiles were evaporated again. The process was repeated 3 times, after which the solvents were evaporated to dryness. The product was precipitated from cold diethyl ether and the precipitated peptide was isolated by centrifugation and lyophilized. A white powder was obtained (89% yield).

ESI-MS: $[M+H]^+$, calculated for $C_{48}H_{49}N_6O_{10}$ 869.34, found: 869.3. $[M+Na]^+$ calculated for $C_{48}H_{48}N_6NaO_{10}$ 891.34, found 891.4.

^{1}H-NMR (DMSO-d6, 300 MHz): δ 12.02 (bs, COOH), 8.44-8.30 (m, 2H), 8.28 – 7.89 (m, 11H), 7.78 (d, J = 8.0 Hz, 1H), 7.30 (s, 1H, NH_2), 7.11 (dd, J = 21.5, 4.5 Hz, 10H, Ar of Phe), 7.04 (s, 1H, NH_2), 4.44-4.36 (m, 2H, H_α, Phe), 4.25-4-16 (m, 4H, overlapping signal H_α Glu and Gly), 3.72-3.71 (d, J = 4.1 Hz, 2H, Pyrene), 3.01-2.88 (m, 2H, H_β, Phe), 2.82-2.63 (m, 2H, H_β, Phe), 2.20-2.09 (m, 4H, m, H_γ, Glu), 1.86-1.58 (m, 4H, H_β, Glu) ppm.

^{13}C-NMR (DMSO-d6, 75 MHz) δ 174.79, 174.73, 173.42, 171.91, 171.86, 171.47, 169.85, 138.54, 138.50, 131.63, 131.52, 131.18, 130.58, 129.97, 129.85, 129.49, 128.84, 128.78, 128.19, 128.08, 127.64, 126.98, 125.87, 125.72, 125.56, 124.90, 124.73, 54.73, 54.49, 53.06, 52.72, 43.10, 38.30, 37.72, 30.83, 28.13, 20.02.

FT-IR (KBr): $\tilde{\nu}$ (cm^{-1}) = 3403, 3311, 3030, 2927, 1737, 1722, 1659, 1652, 1615, 1540, 14898, 1453, 1443, 1409, 1272, 1185, 1170, 846, 792, 744, 699, 646, 496.

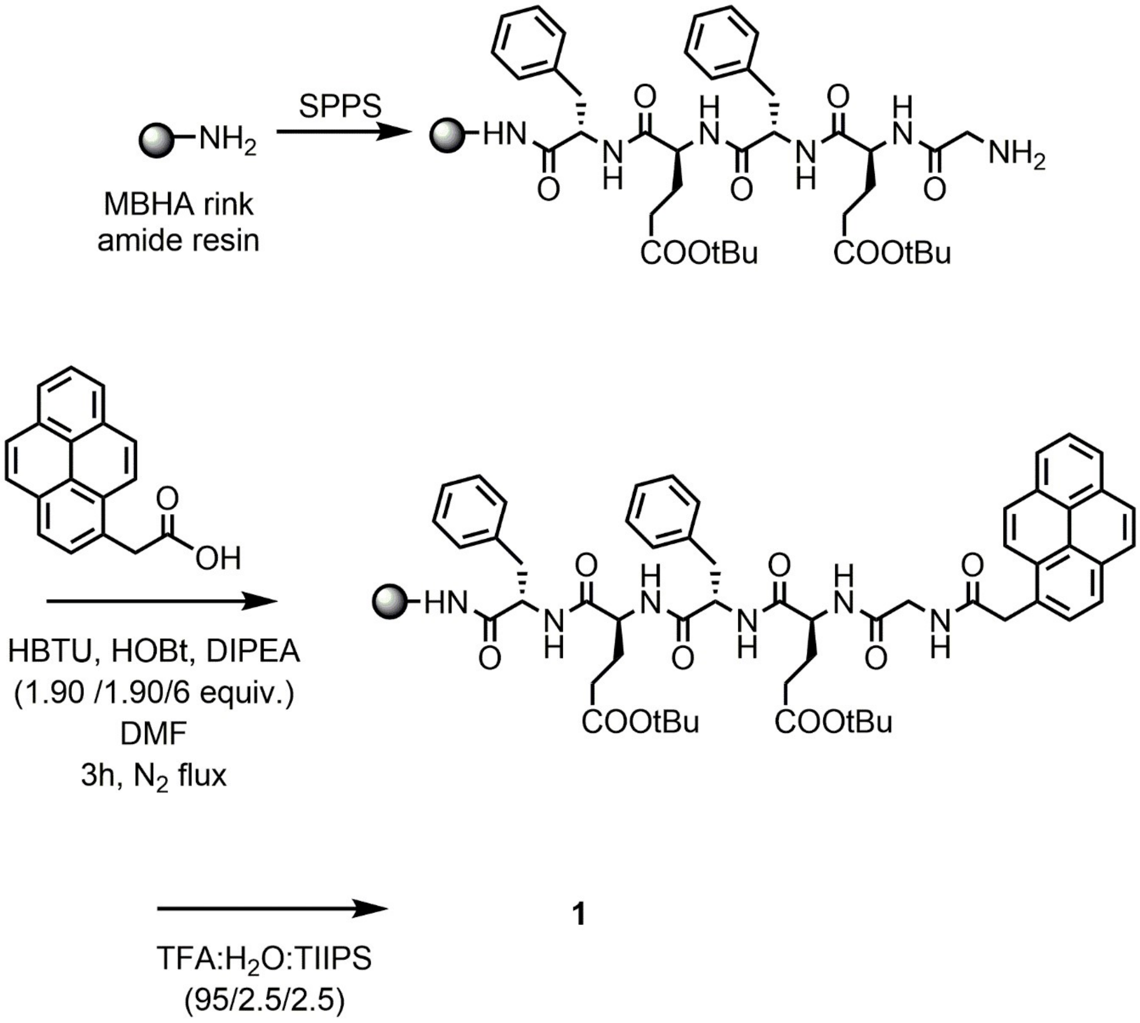

Scheme S1: synthesis of **1**.

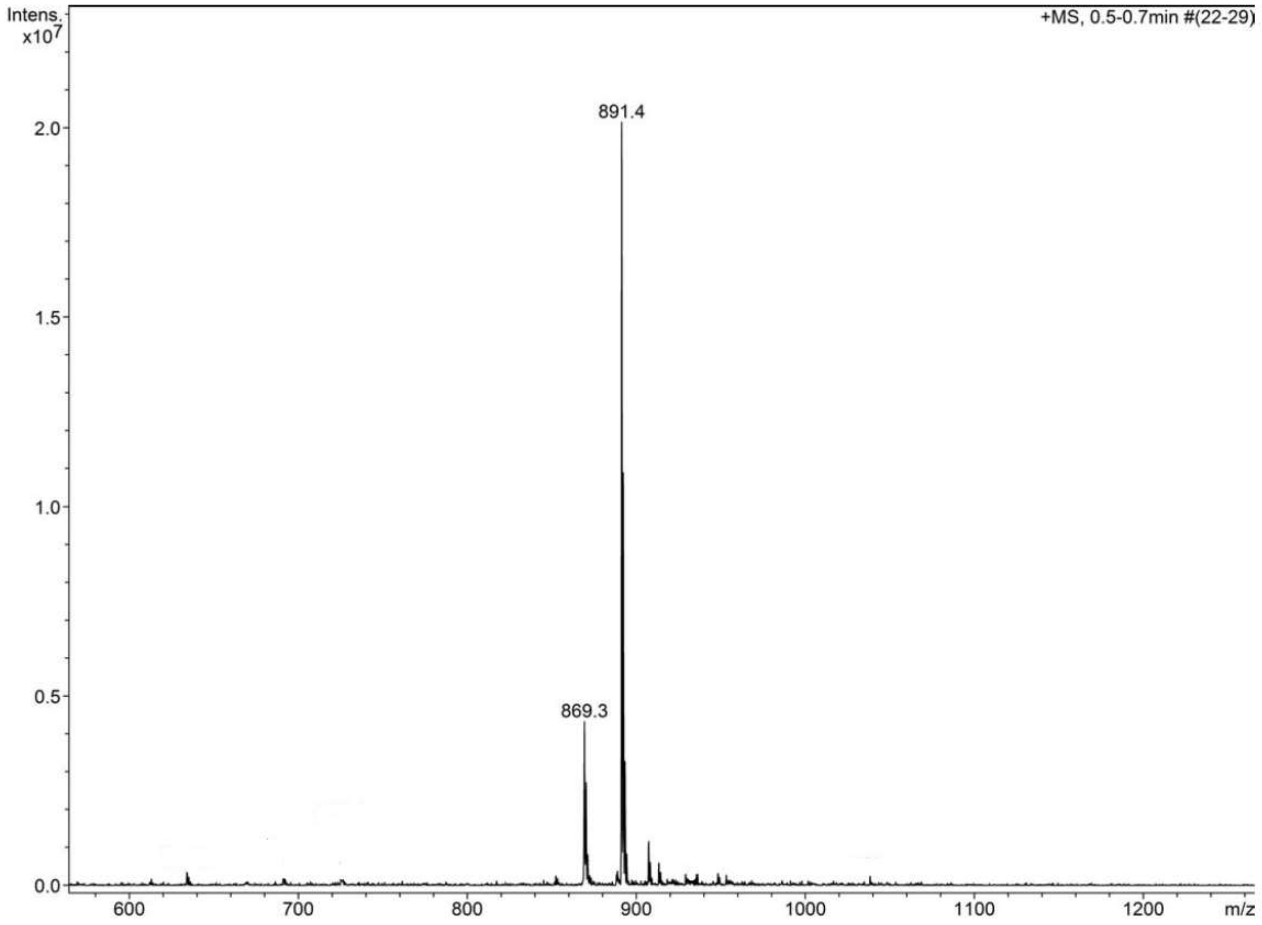

Figure S1: ESI-MS compound **1** in MeOH.

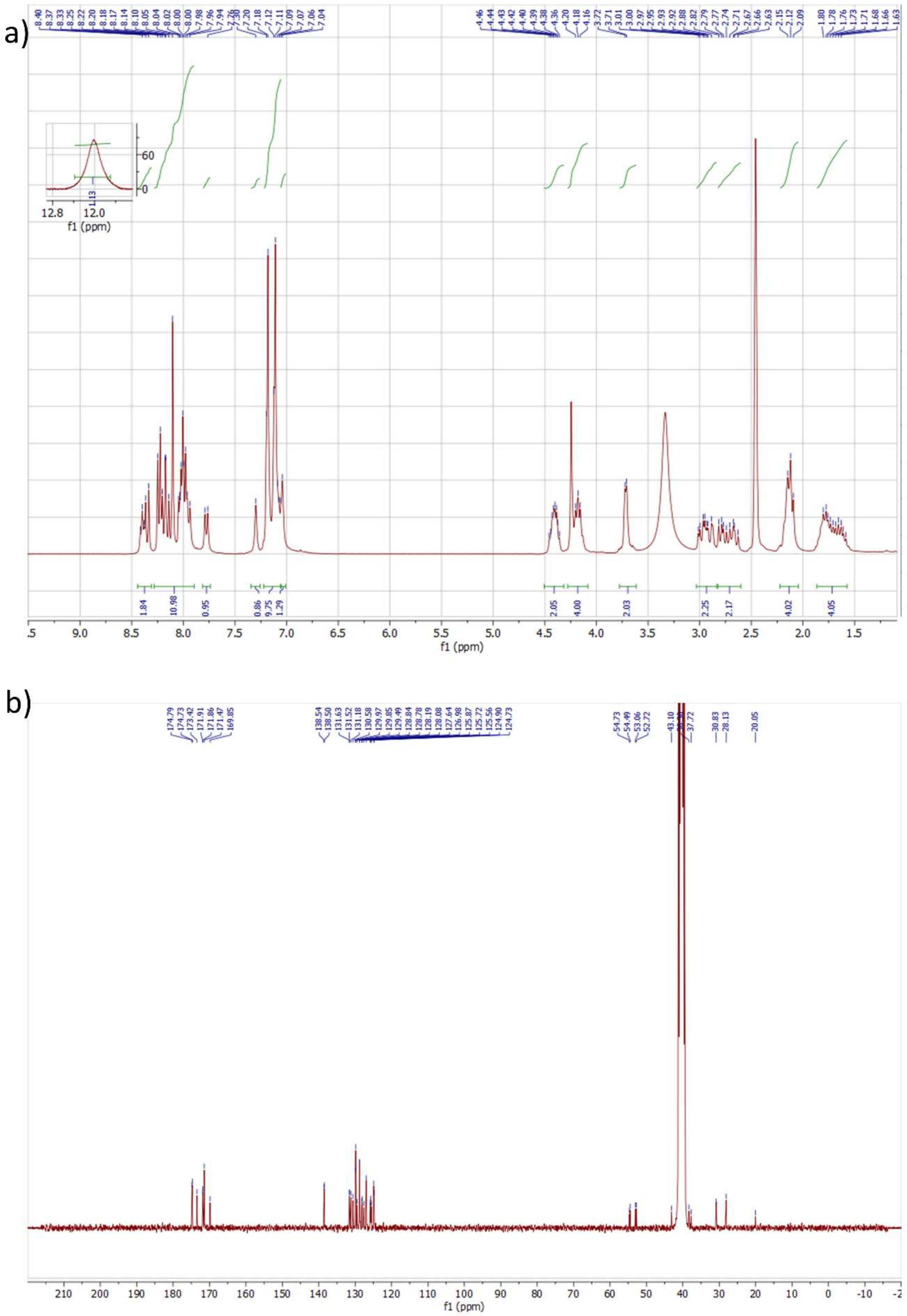

Figure S2: a) ^{1}H NMR (DMSO-d_6, 300 MHz) of **1; b)** ^{13}C-NMR (DMSO-d6, 75 MHz).

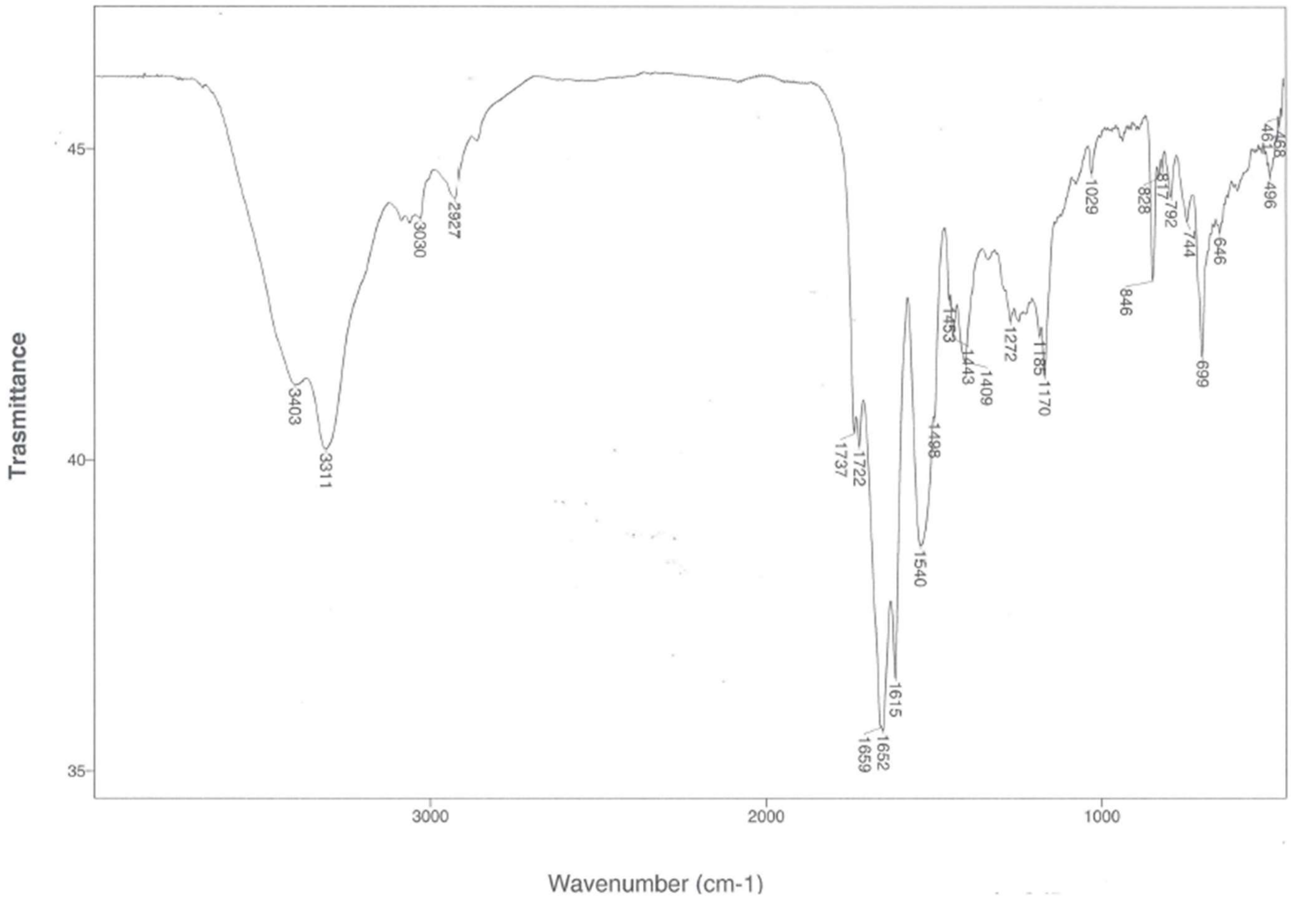

Figure S3: FT-IR spectrum of **1** (KBr disk).

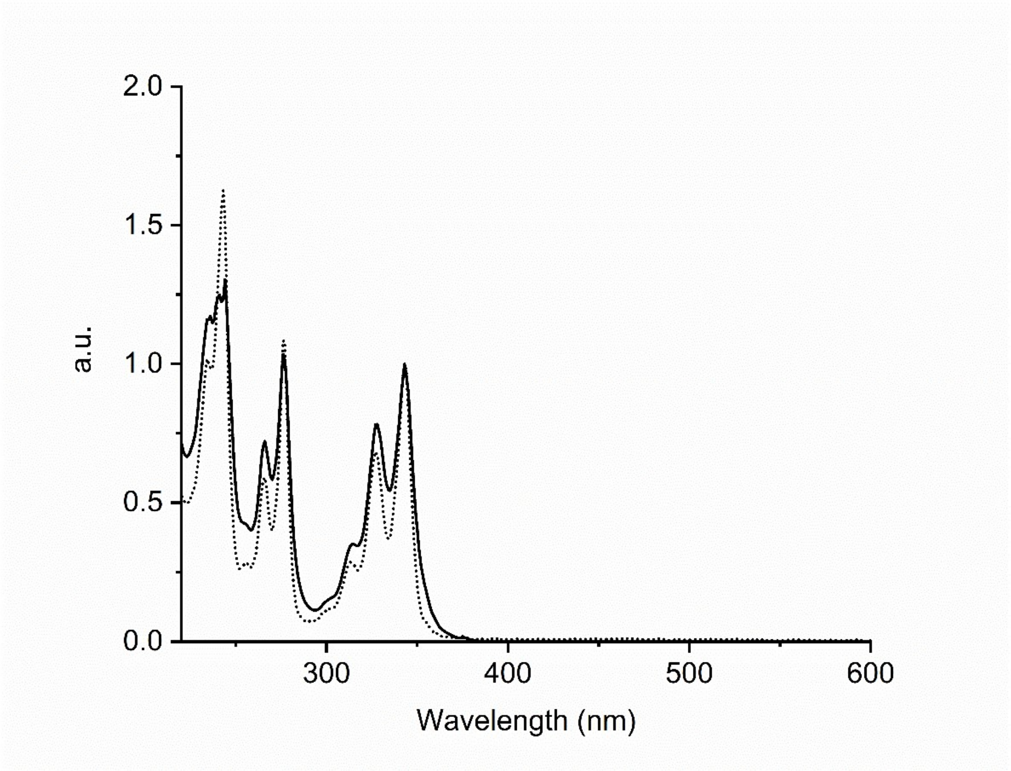

Figure S4. Normalized absorption spectra of **1** in basic water at 0.5% (solid line) and at 0.005% (dot line).

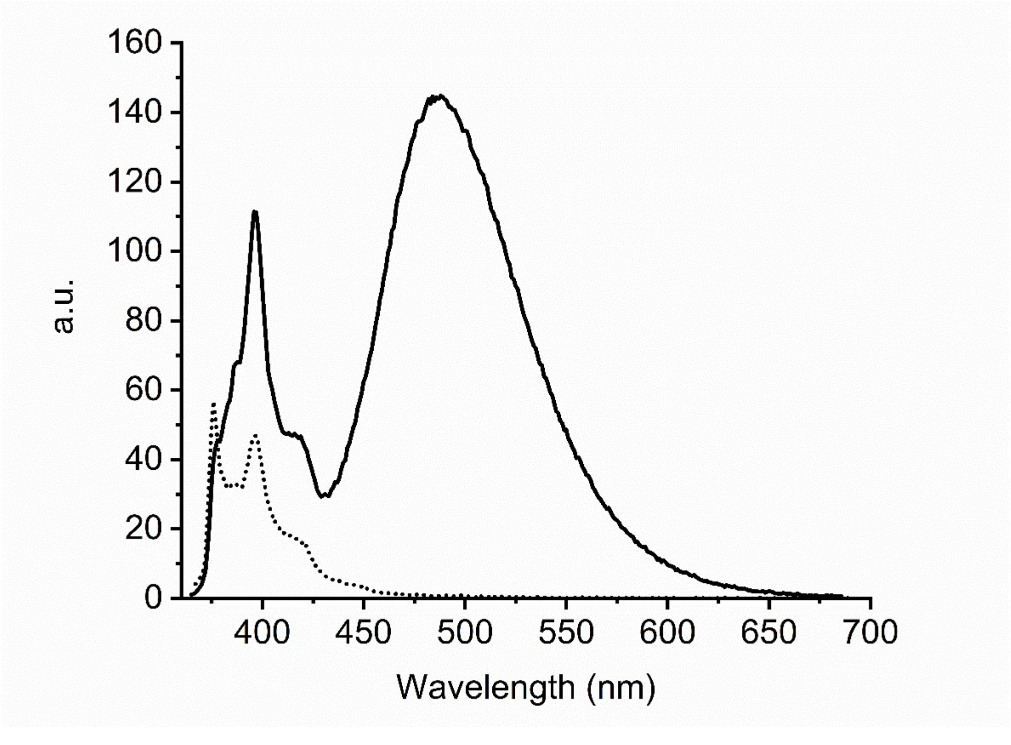

Figure S5. Non-normalized emission spectra of **1** in basic water at 0.5% (solid line) and at 0.005% (dot line).

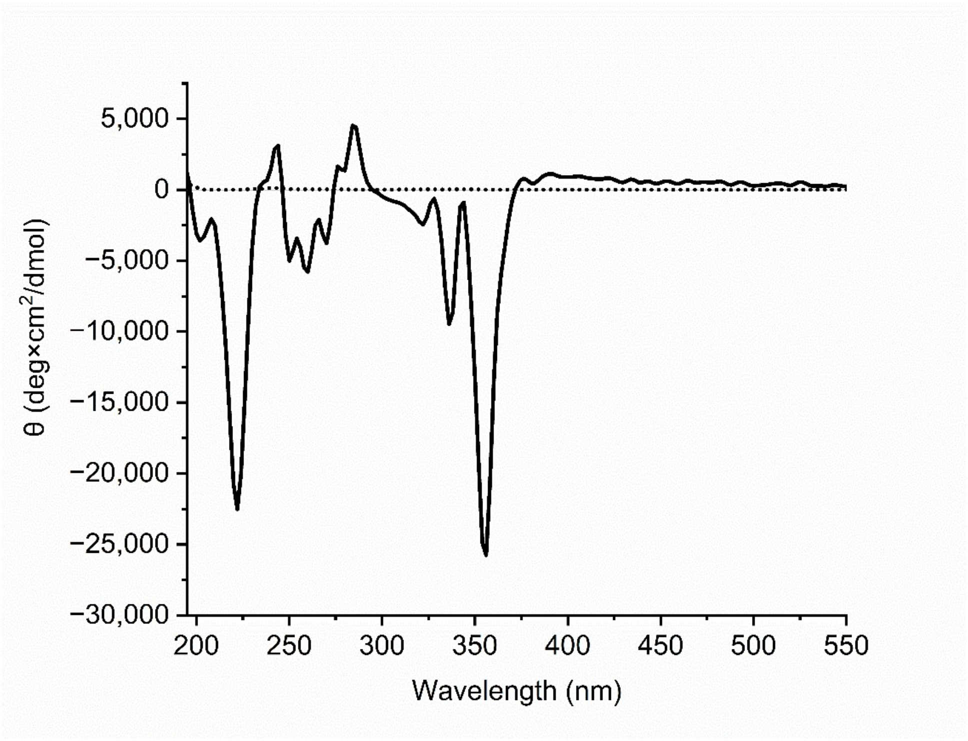

Figure S6: CD spectra of **1** in basic water at 0.5% (solid line) and at 0.005% (dot line).

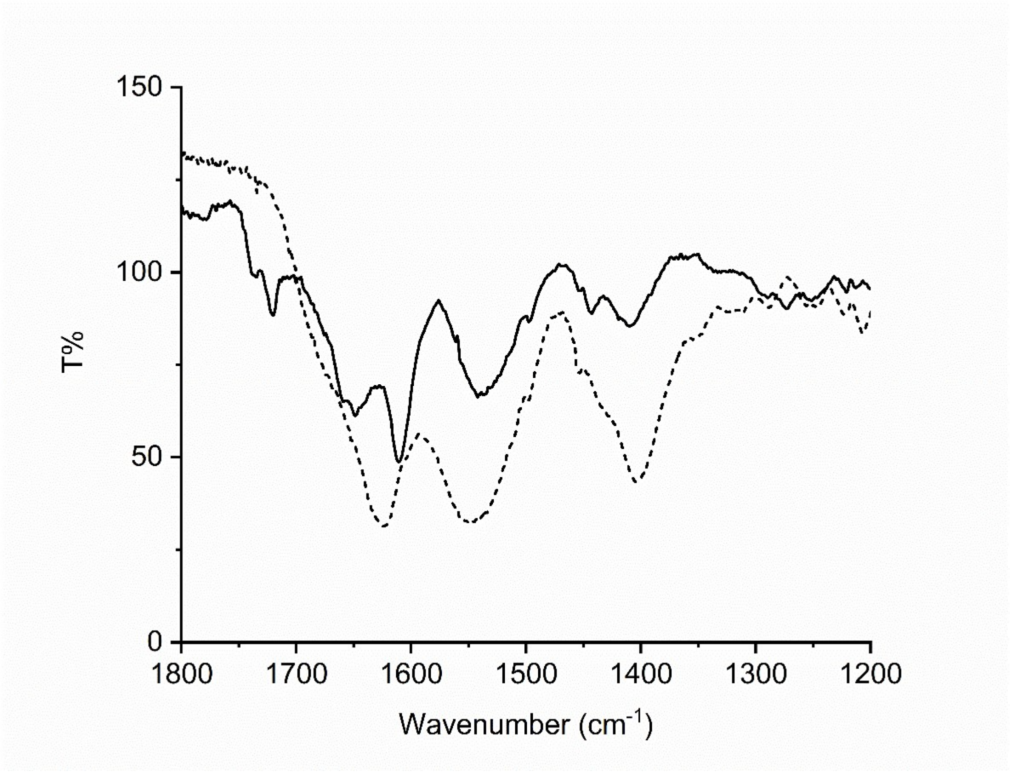

Figure S7: ATR-FTIR spectrum of **1** (solid line) and the deprotonated sample (dot line).

PREPARATION AND CHARACTERIZATION OF GELS

pH triggered gelation. A known amount of **1** was introduced in a 4 mL vial. Then 900 µL of milliQ water were added. NaOH 1M was added in portions of 10 µL till a clear solution through sonication was obtained followed by addition of water to a final volume of 1 mL. Then HCl 1M was added in portions. Gel formation was assessed by the vial inversion test.

Alkali metal salt induced gelation. A known amount of **1** was introduced in a 4 mL vial. Then 900 µL of milliQ water were added. NaOH 1M was added in portions of 10 µL till a clear solution was obtained (sonication) followed by addition of water to a final volume of 1 mL. Then a solution of alkali salt 1M was added in portions of 10 µL till gel formation was observed. Gel formation was assessed by the vial inversion test.

Bi-and-trivalent metal salts induced gelation. A known amount of **1** was introduced in a 4 mL vial. Then 900 µL of milliQ water were added. NaOH 1M was added in portions of 10 µL till a clear solution was obtained (sonication) followed by addition of water to a final volume of 1 mL. Then a solution of salt 0.05M was added in proportion 1:1 with the compound **1**. Gel formation was assessed by the vial inversion test.

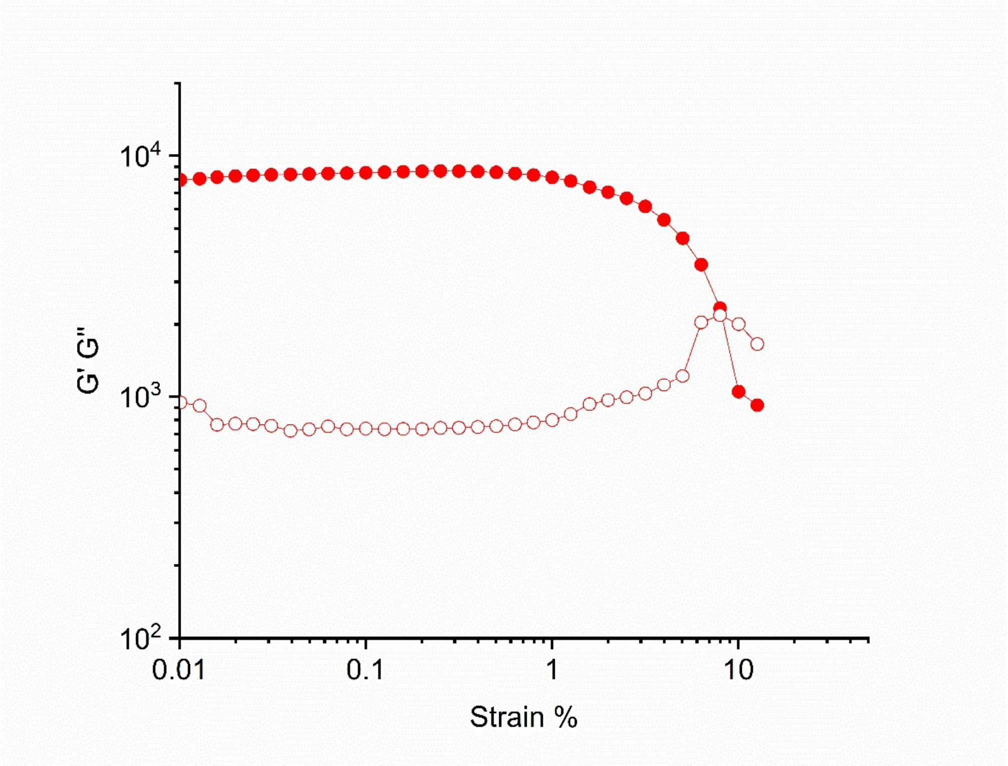

Figure S8: Amplitude sweep of **1-HCl** gel.

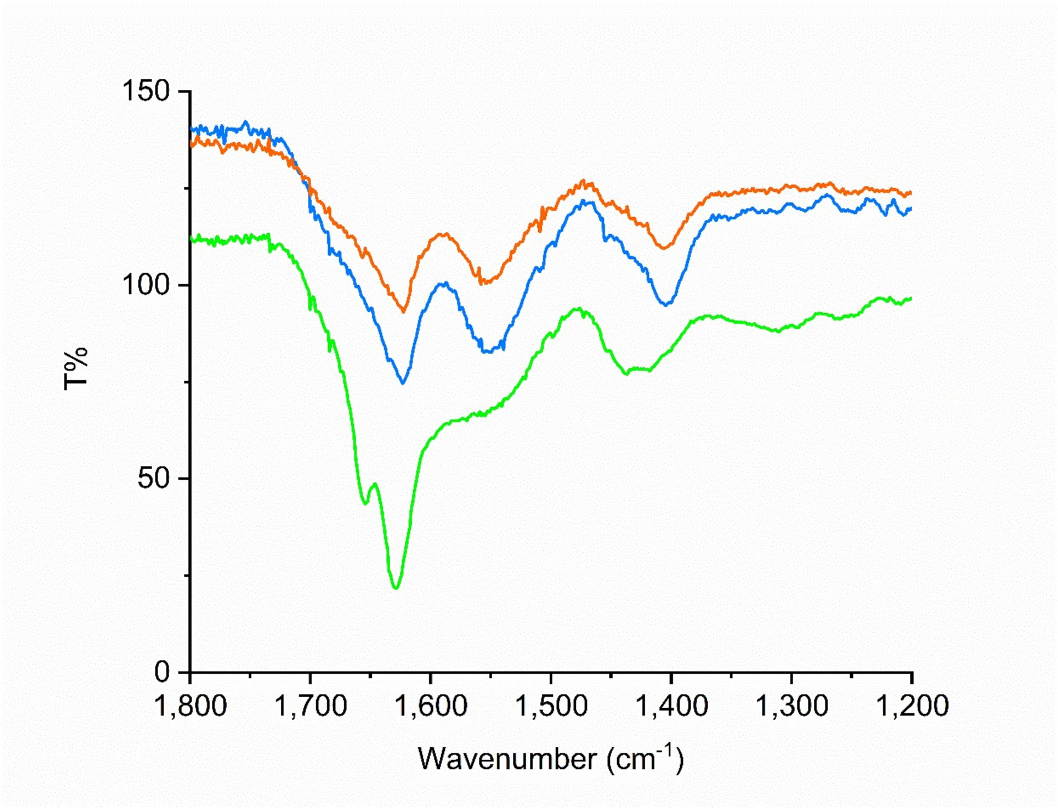

Figure S9: ATR-FTIR spectrum of monovalent induced hydrogels; **1-Li** (green), **1-Na** (blue), **1-K** (orange).

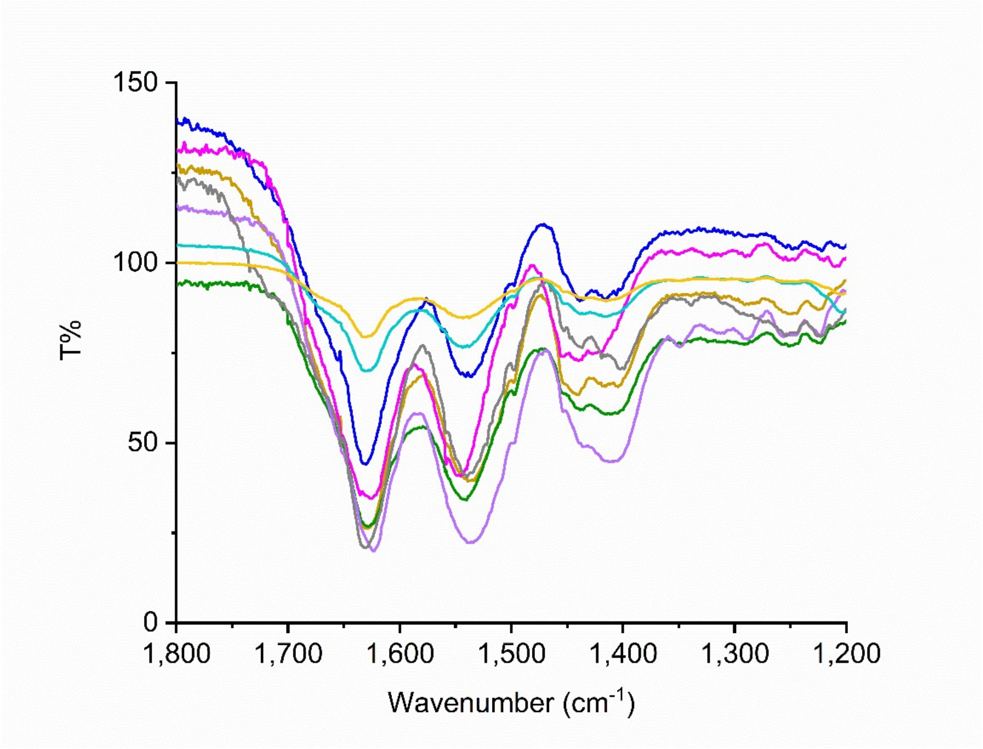

Figure S10: ATR-FTIR spectrum of di-and-trivalent metallogels; **1-Ca** (magenta), **1-Cr** (ochre), **1-Fe** (grey), **1-Co** (yellow), **1-Ni** (light blue), **1-Cu** (blue), **1-Zn** (green), **1-Cd** (violet).

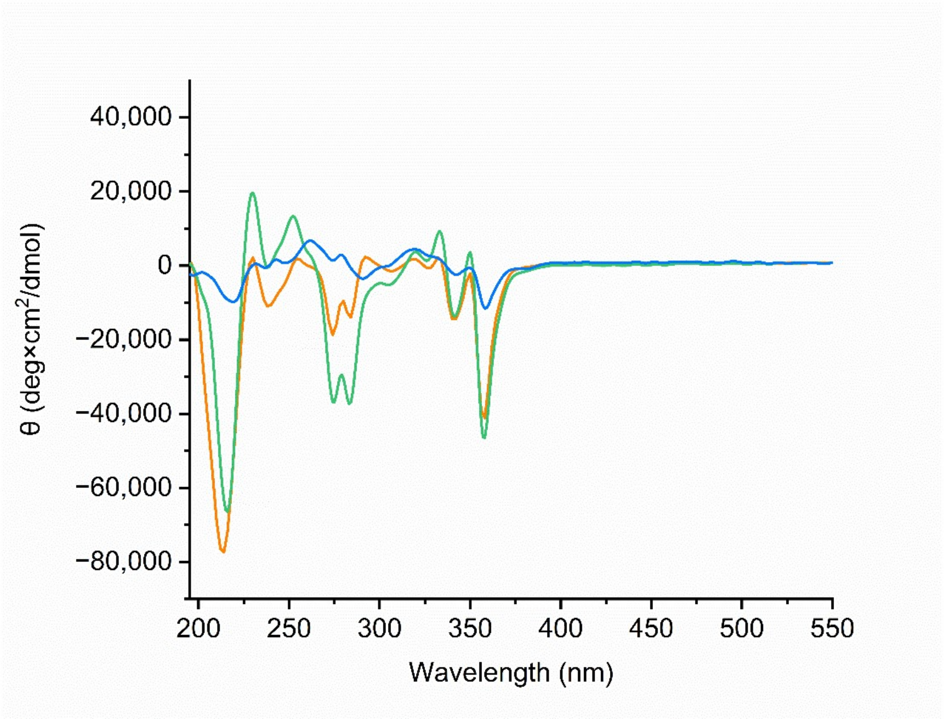

Figure S11: CD spectra of monovalent hydrogels; **1-Li** (green), **1-Na** (blue), **1-K** (orange).

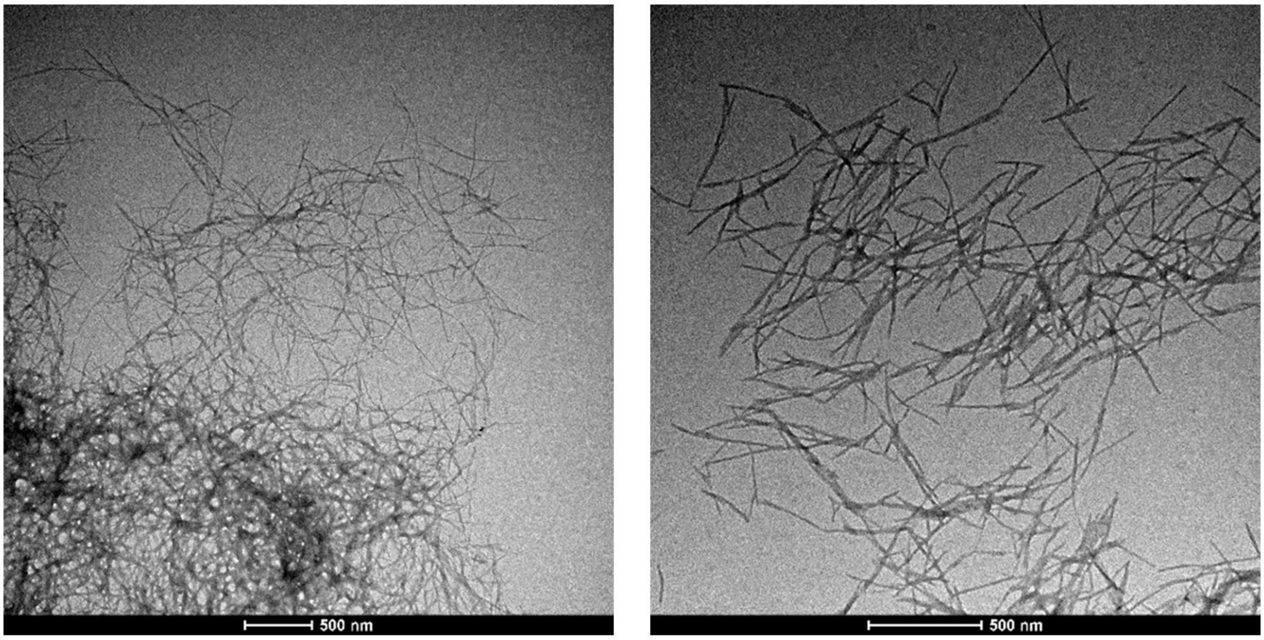

Figure S12: TEM images of **1-HCl** gel.

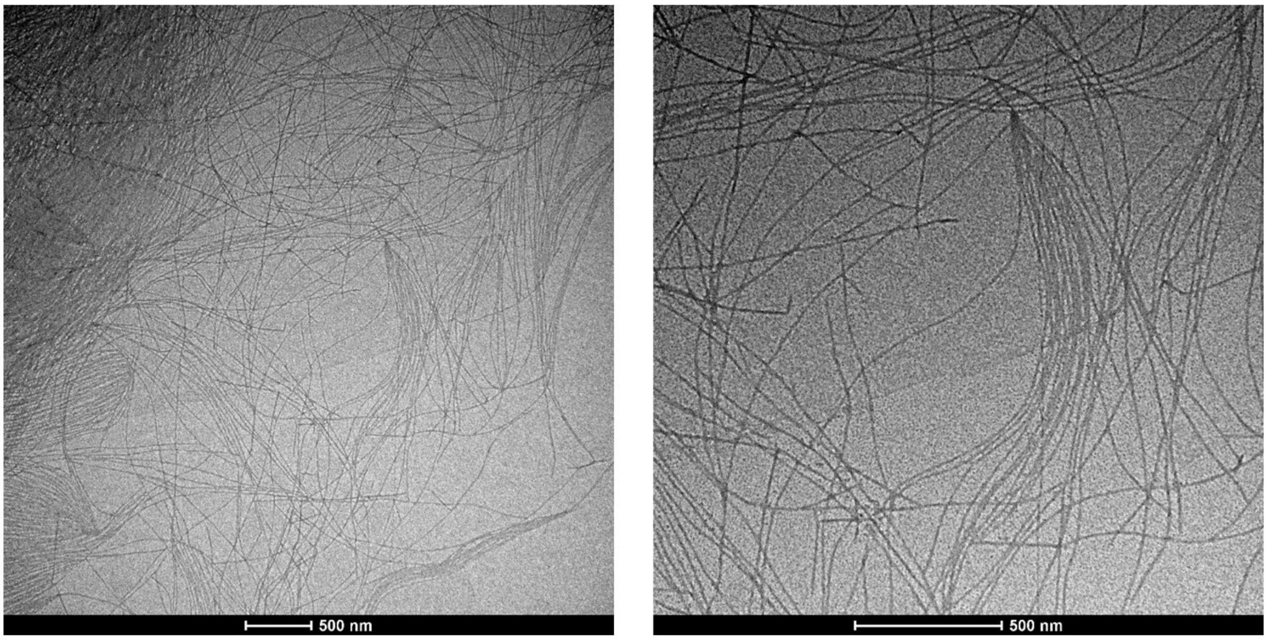

Figure S13: TEM images of **1-Li**.

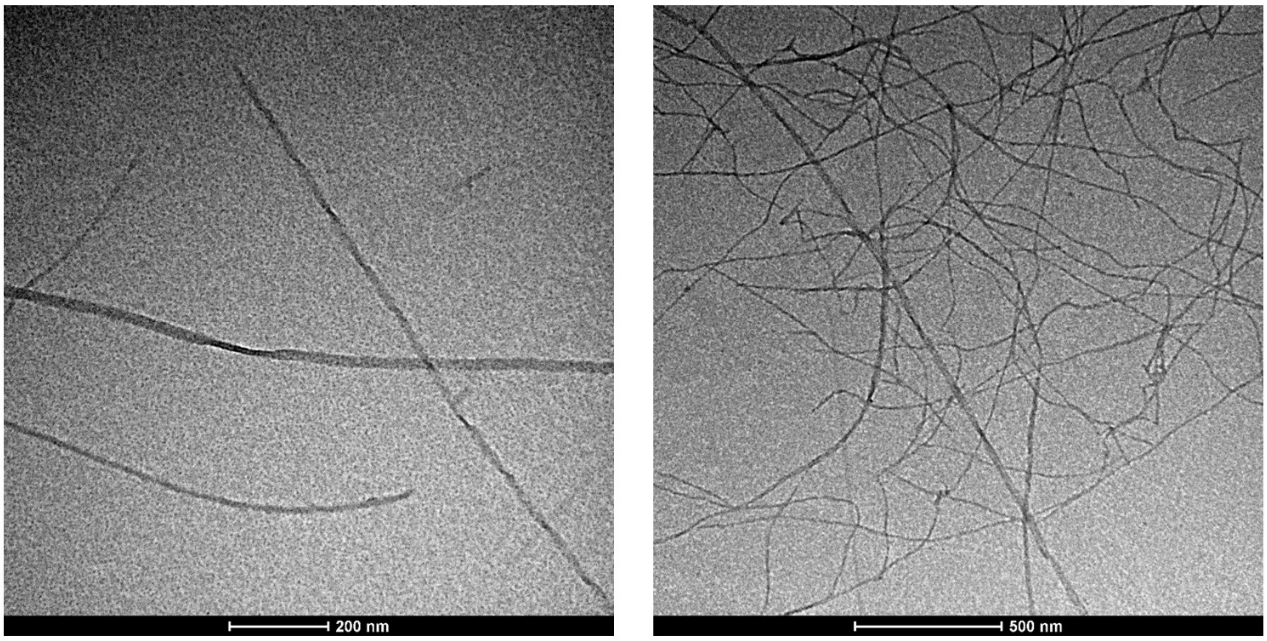

Figure S14: TEM images of **1-Na**.

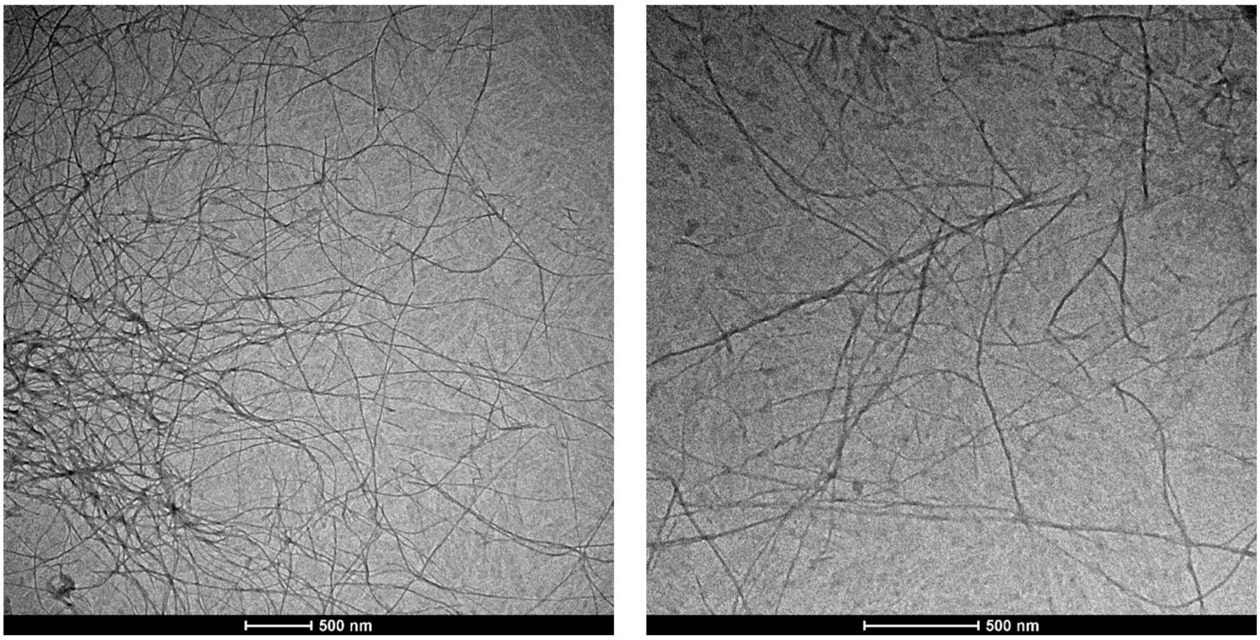

Figure S15: TEM images of **1-K**.

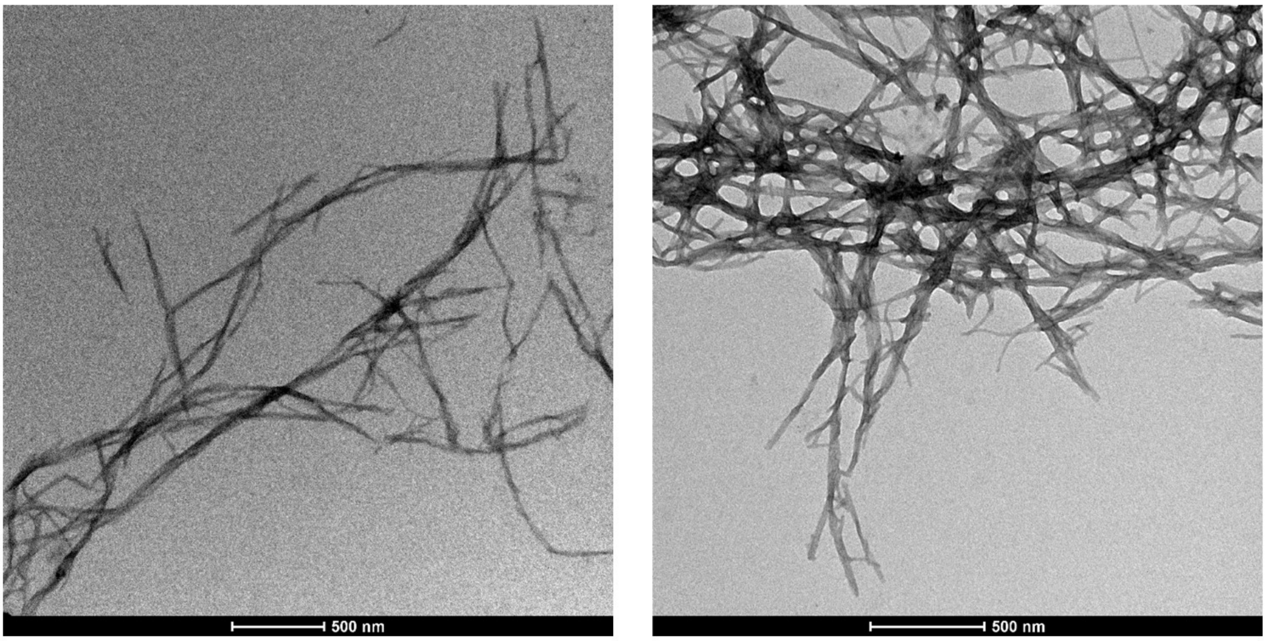

Figure 16: TEM images of **1-Ca**.

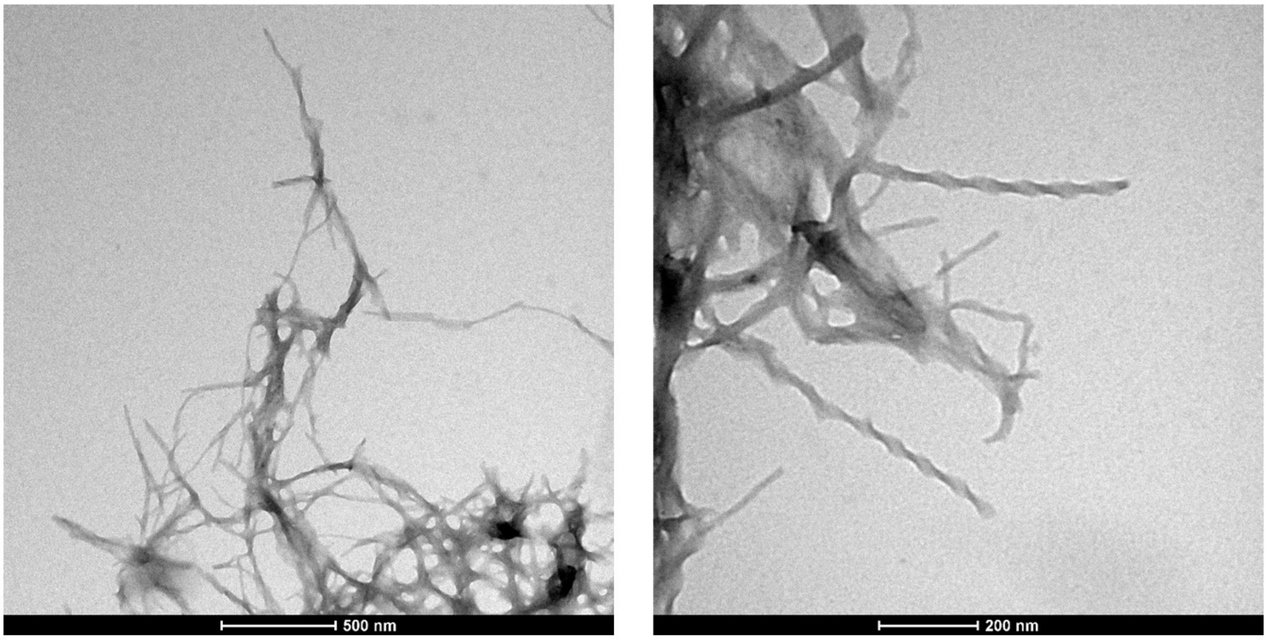

Figure S17: TEM images of **1-Cd**.

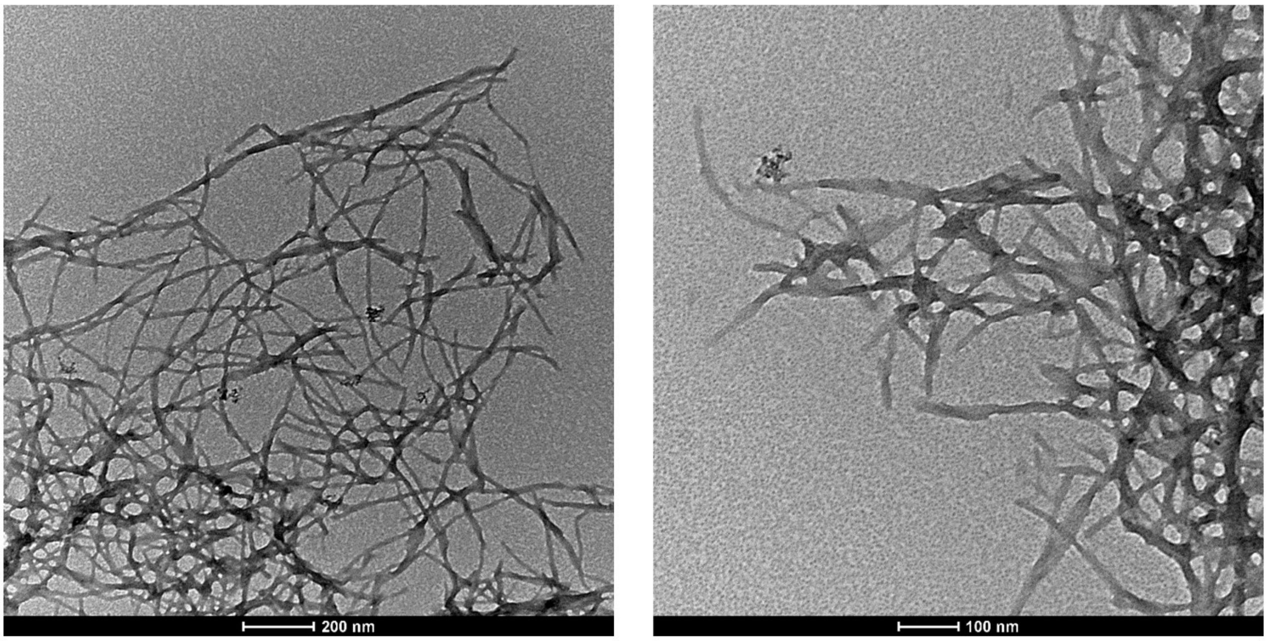

Figure S18: TEM images of **1-Co**.

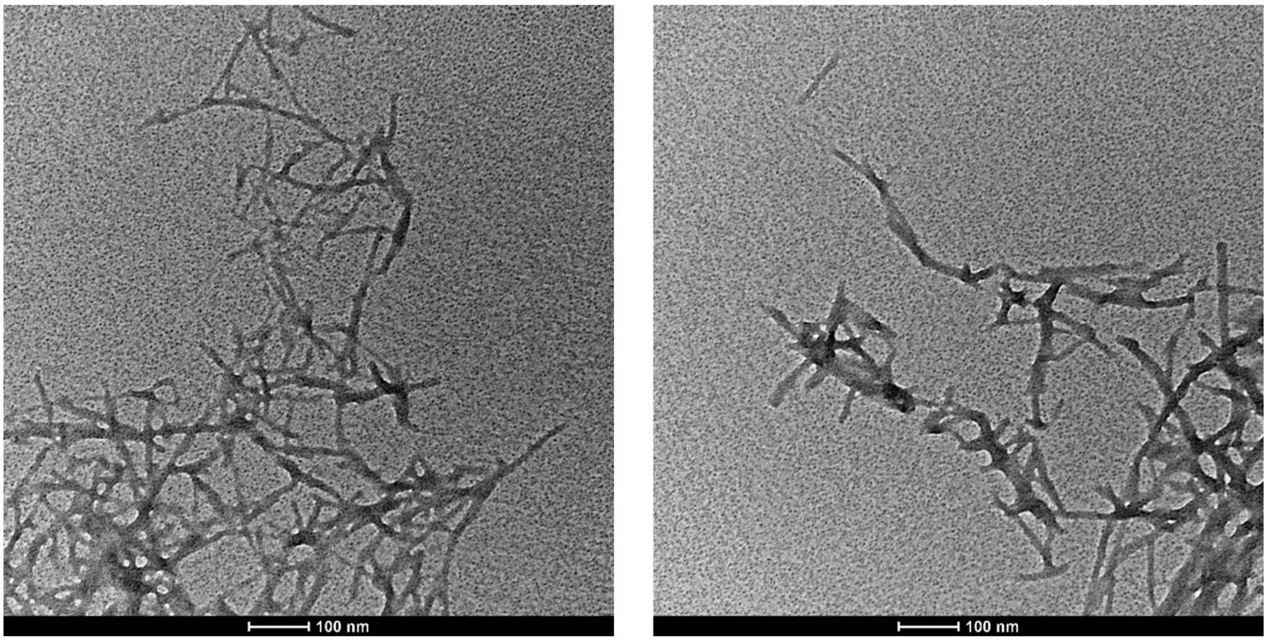

Figure S19: TEM images of **1-Ni**.

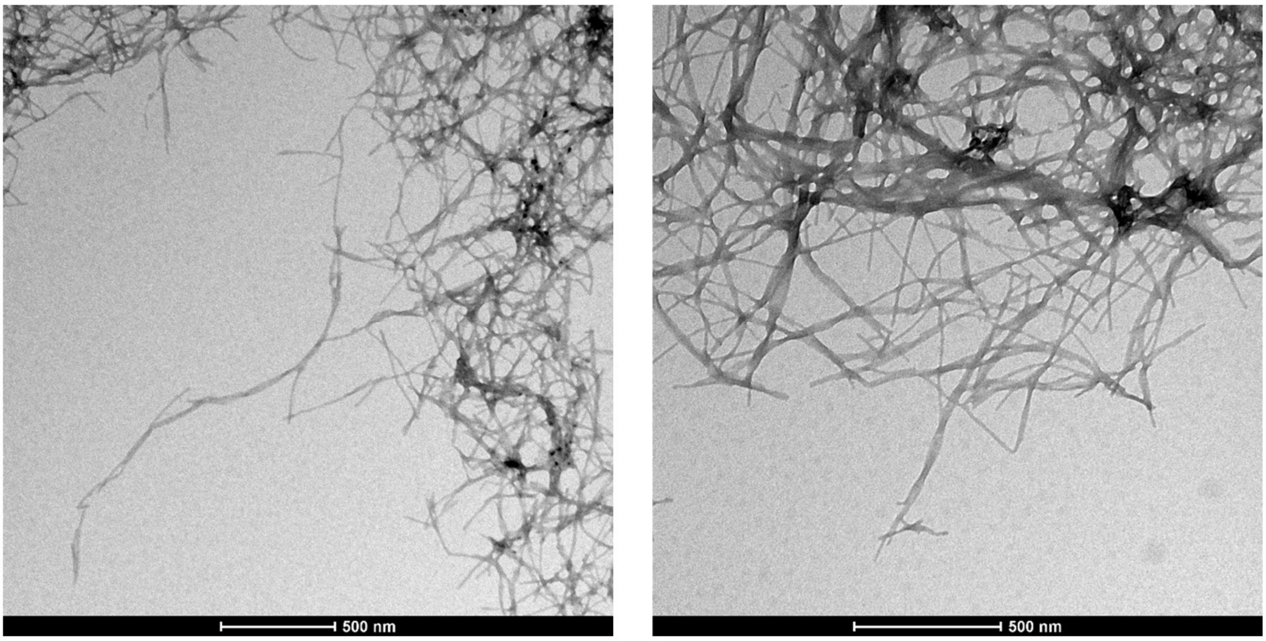

Figure S20: TEM images of **1-Zn**.

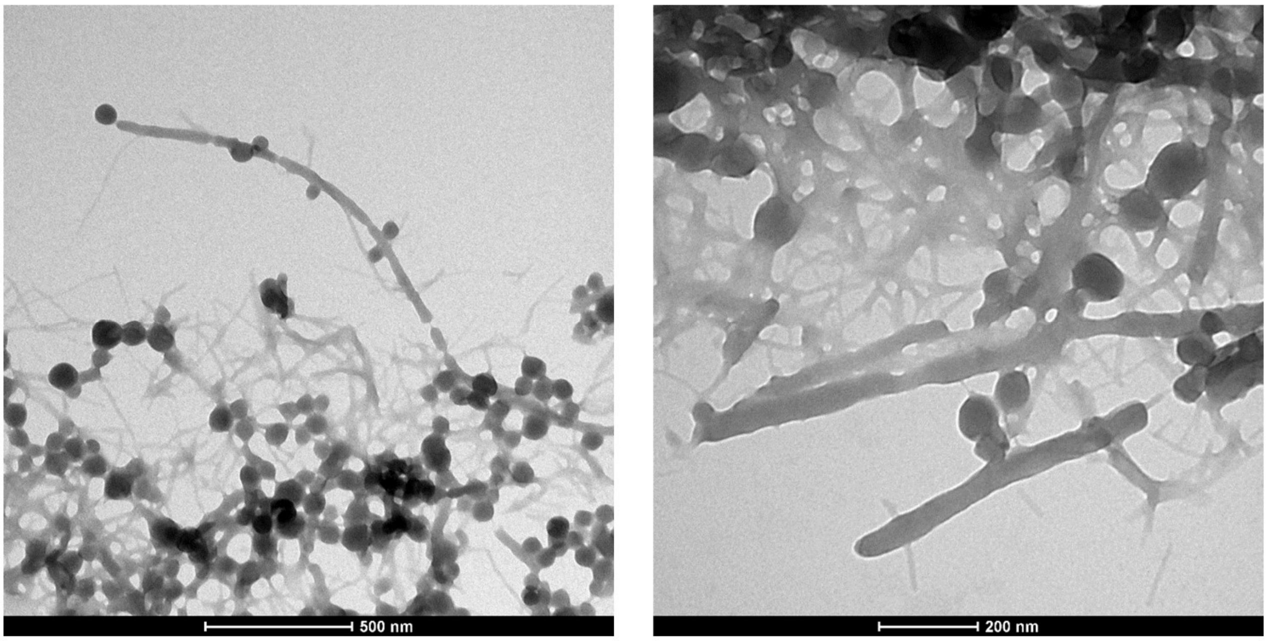

Figure S21: TEM images of **1-Cu**.

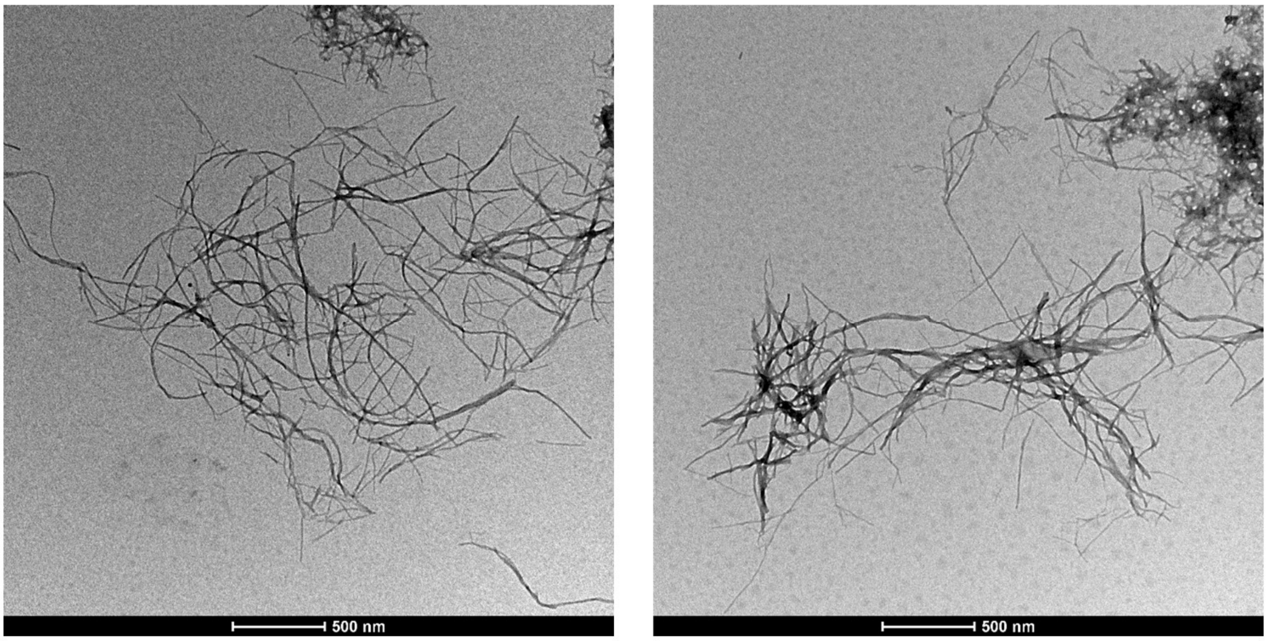

Figure S22: TEM images of **1-Cr**.

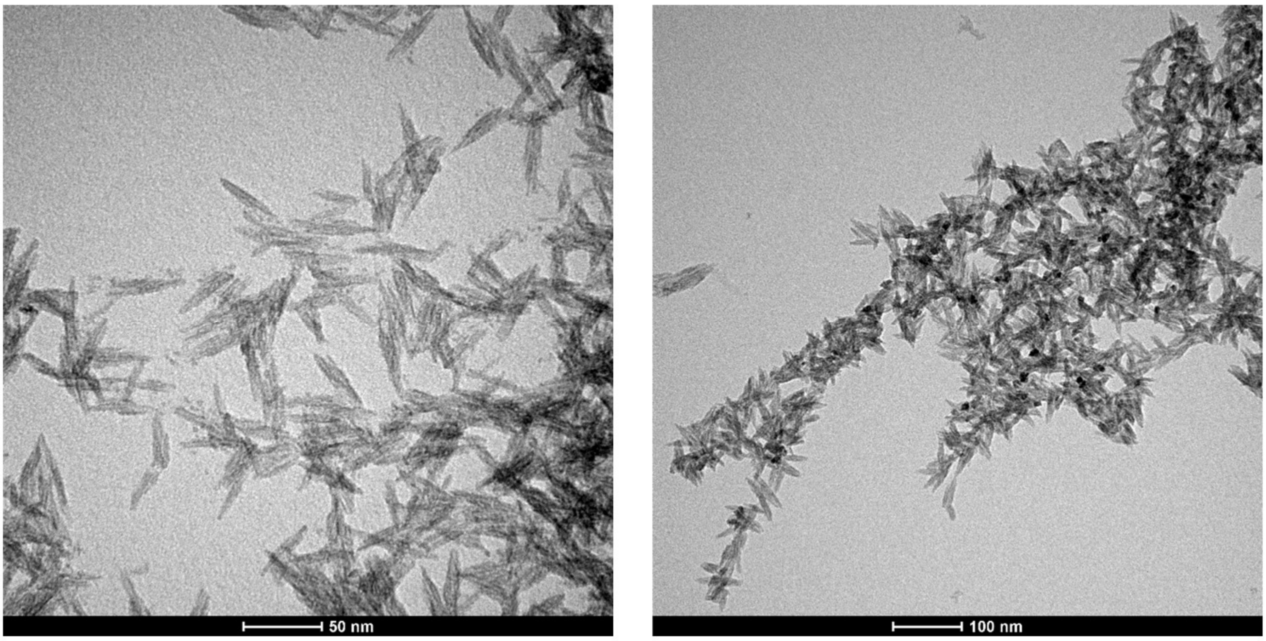

Figure S23: TEM images of **1-Fe**.

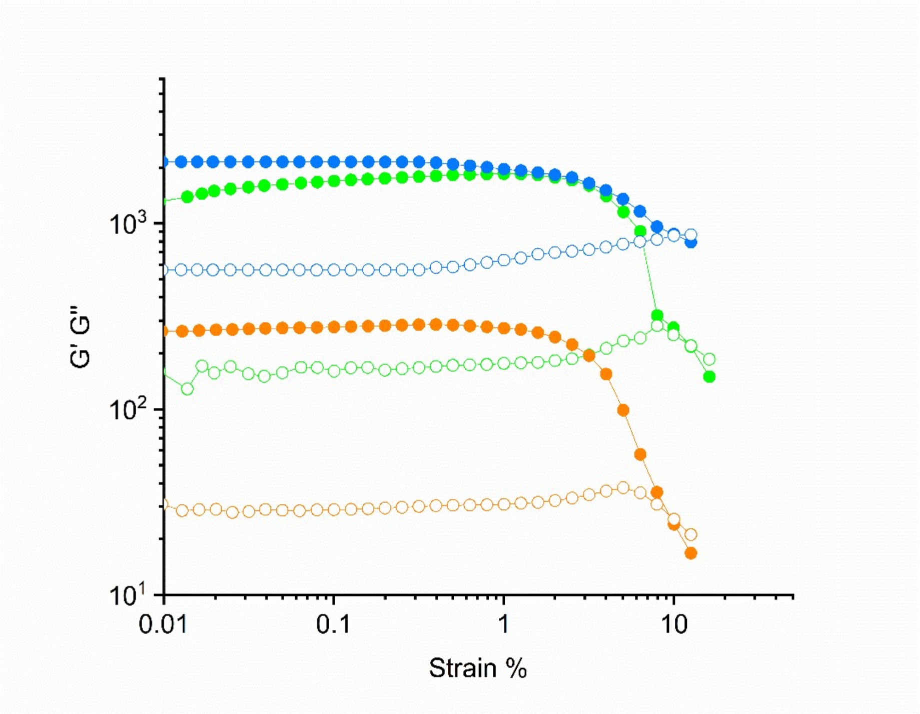

Figure S24: Amplitude sweep of monovalent hydrogels; **1-Li** (green), **1-Na** (blue), **1-K** (orange).

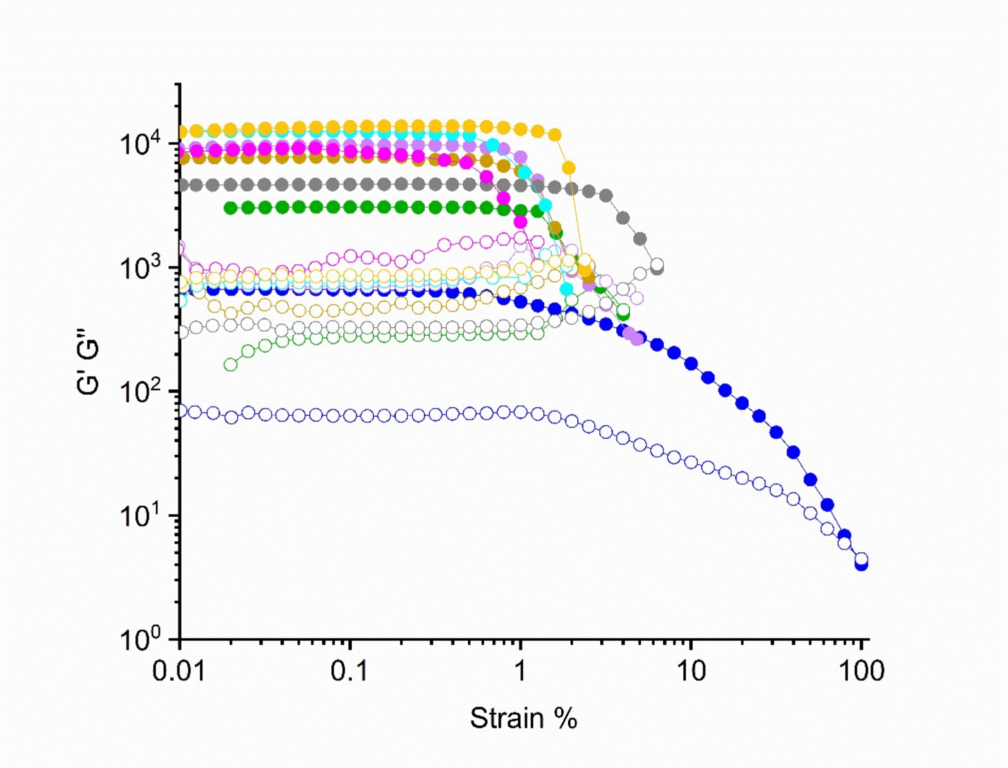

Figure S25: Amplitude sweep of metallogels; ; **1-Ca** (magenta), **1-Cr** (ochre), **1-Fe** (grey), **1-Co** (yellow), **1-Ni** (light blue), **1-Cu** (blue), **1-Zn** (green), **1-Cd** (violet).

Sample	G’ (Pa)	G’’(Pa)	Crossover point G’=G’’ (% strain)
1-HCl	6,702 ± 20	628 ± 4	8
1-Li	1,499 ± 11	116.9 ± 0.8	12.6
1-Na	1,481 ± 27	502 ± 11	10.4
1-K	206 ± 1	26.3 ± 0.3	9.3
1-Ca	8,814 ± 66	934 ± 14	1.1
1-Zn	2,632 ± 19	274 ± 4	2.9
1-Cd	9,679 ± 70	797 ± 14	1.8
1-Ni	11,444 ± 41	807 ± 12	1.7
1-Co	12,796 ± 46	948 ± 14	2.5
1-Cu	526 ± 1	68 ± 1	100
1-Cr	7,562 ± 8	487 ± 9	2
1-Fe	4,385 ± 16	337 ± 2	6.2

Table T1. Average value of G’ and G’’ and the flow point.

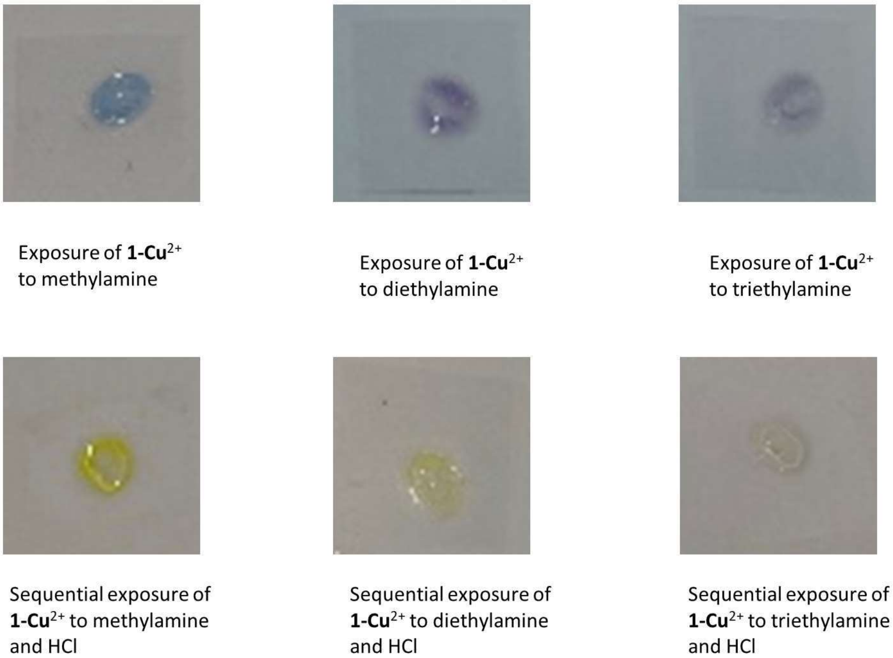

Figure S246. Colorimetric responses of the 1-Cu^{2+} gel to methylamine, diethylammine and triethylammine

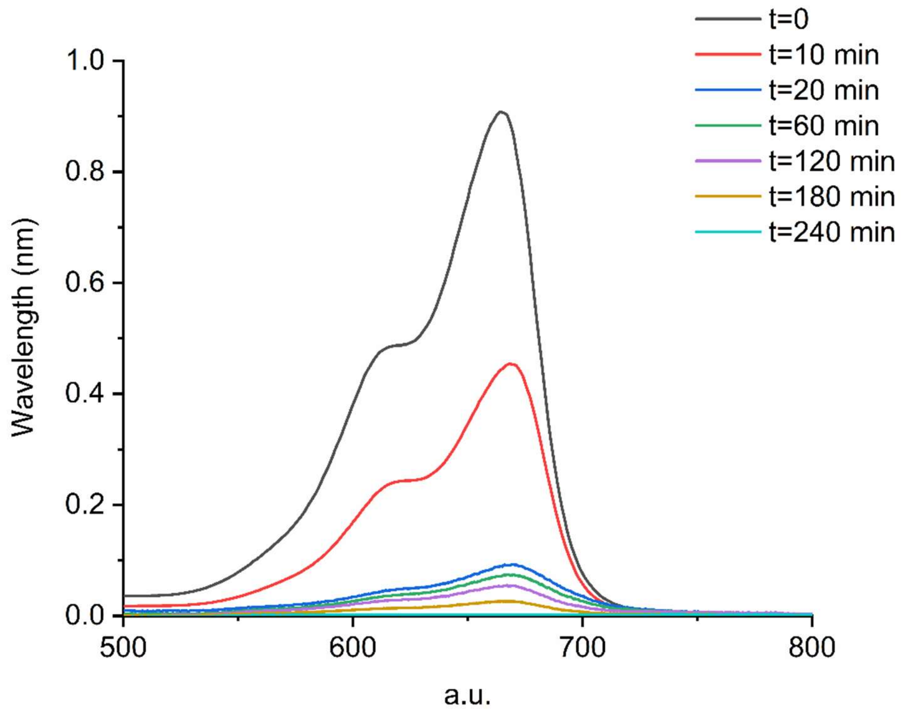

Figure S27: Absorption spectra of methylene blue in presence of **1-Zn** xerogel.

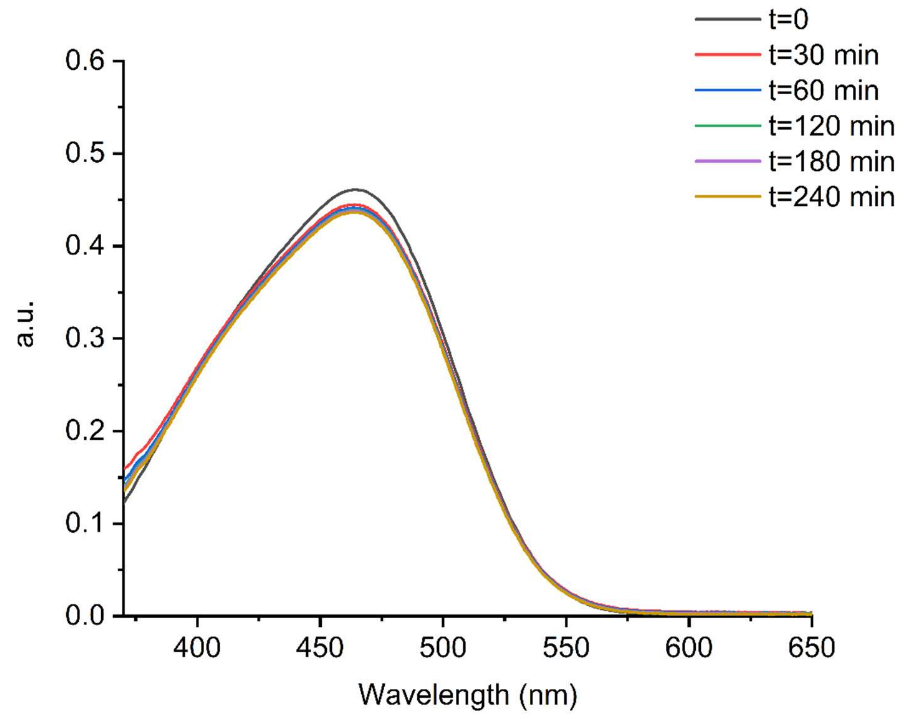

Figure S28: Absorption spectra of methyl orange in presence of **1-Zn** xerogel

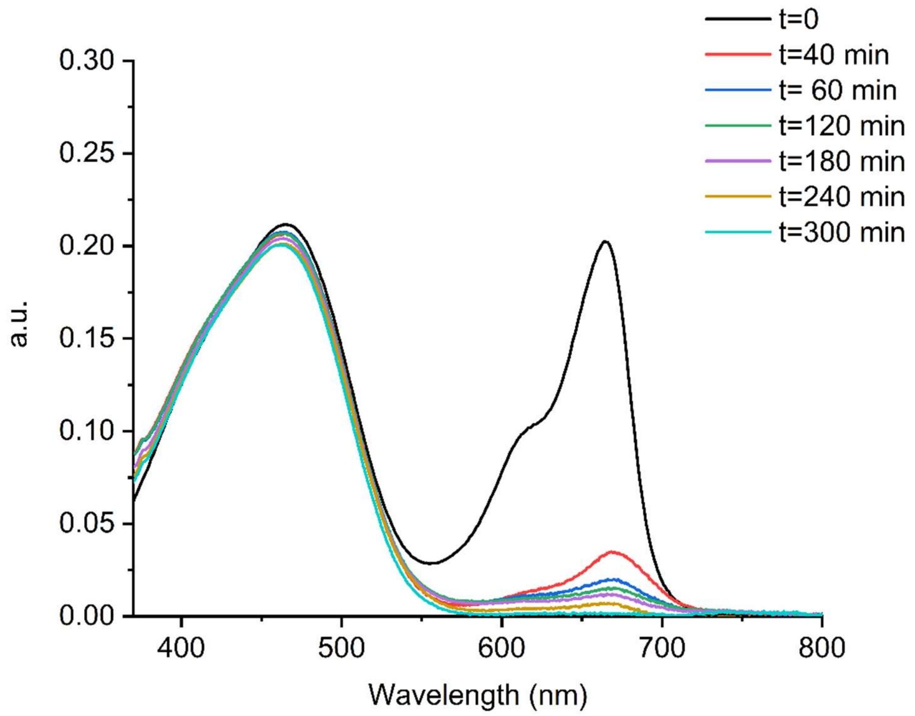

Figure S29: Absorption spectra of methylene blue mixed with methyl orange in presence of **1-Zn** xerogel.

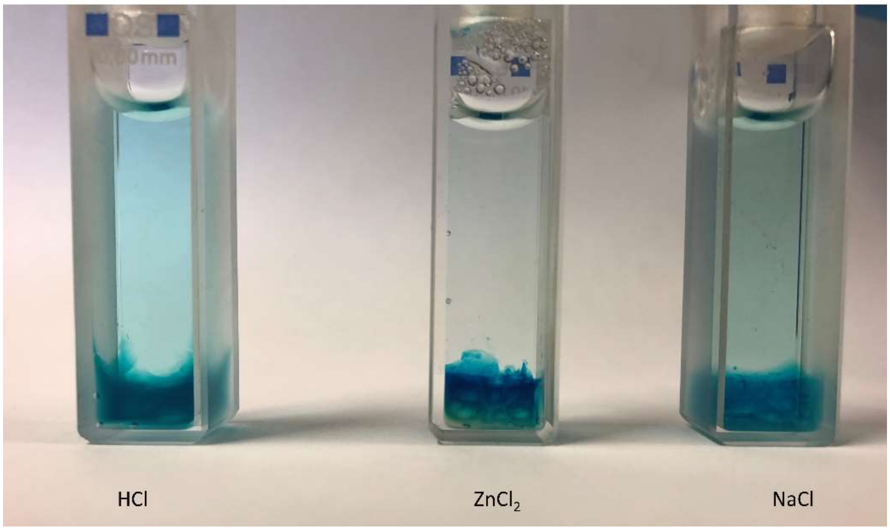

Figure S30: Photo of cuvettes containing hydrogels and absorbed methylene blue.

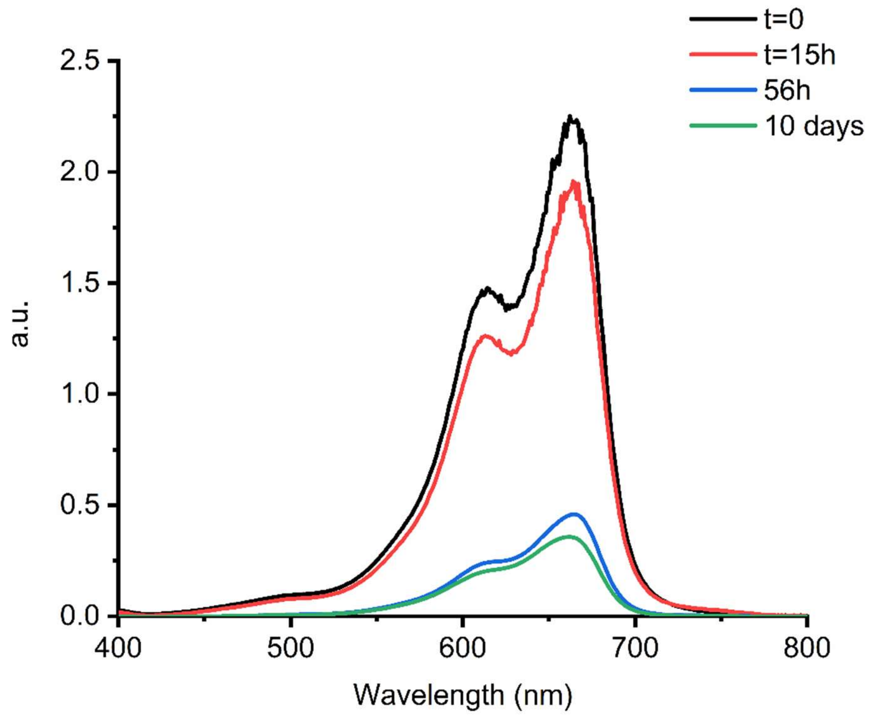

Figure S31: Absorption spectra of methylene in presence of **1-HCl** hydrogel.

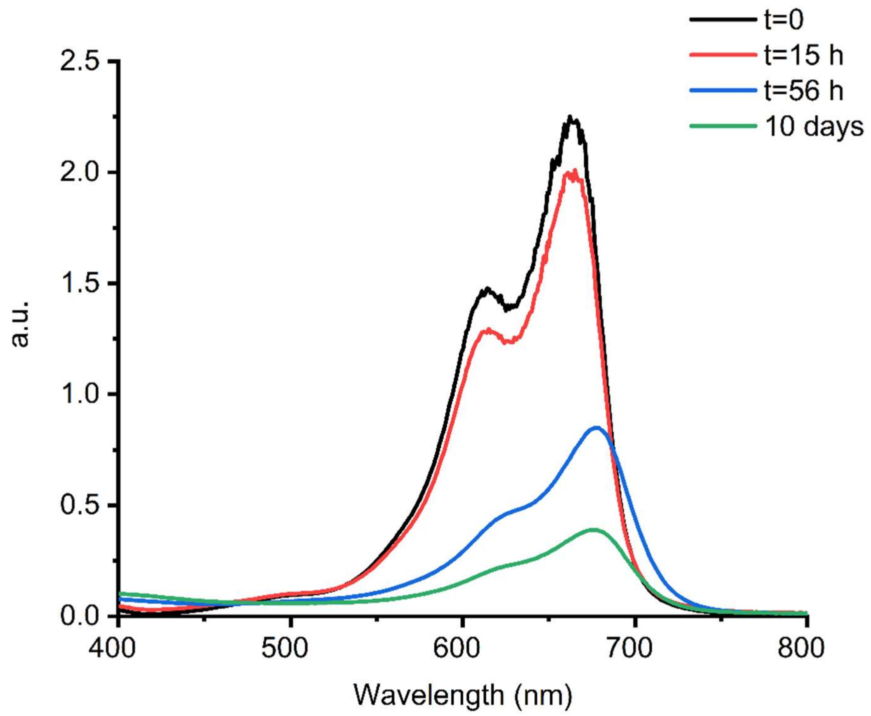

Figure S32: Absorption spectra of methylene in presence of **1-Na** hydrogel.

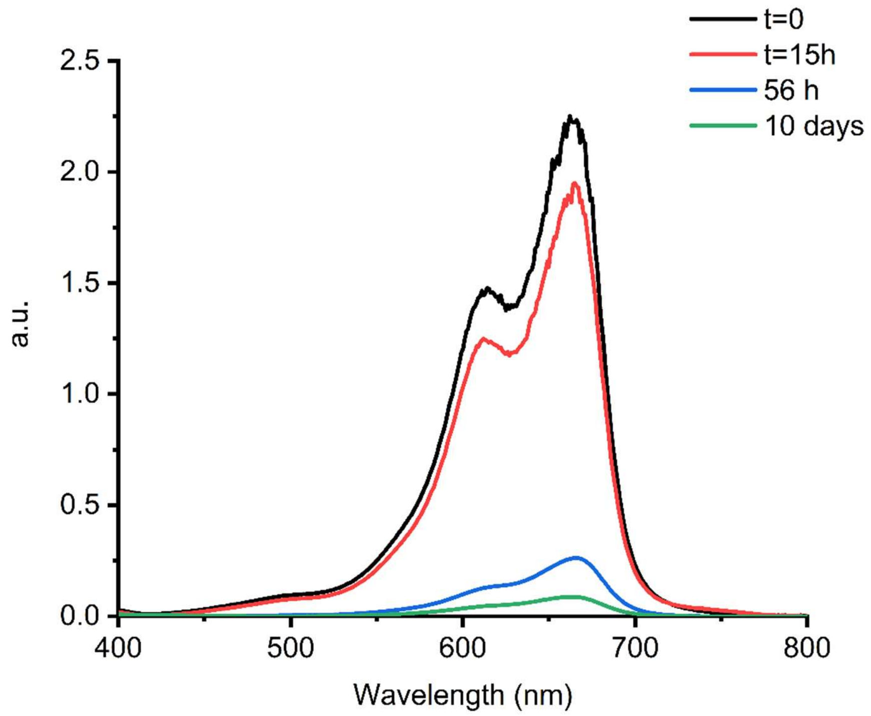

Figure S33: Absorption spectra of methylene in presence of **1-Zn** hydrogel.
